# Gut microbiome depletion aggravates glial-mediated Alzheimer’s disease pathology through inflammatory, metabolic, and proteostatic dysregulation

**DOI:** 10.1080/19490976.2026.2753247

**Published:** 2026-11-08

**Authors:** Kishore Madamanchi, Srinath Gurrala, John Watson, Girish Melkani

**Affiliations:** a Department of Pathology, Division of Molecular and Cellular Pathology, Heersink School of Medicine, University of Alabama at Birmingham, Birmingham, AL, USA; b UAB Nathan Shock Center, Birmingham, AL, USA

**Keywords:** Alzheimer’s disease, gut microbiome, memory impairment, sleep-circadian homeostasis, autophagy defects, gut–brain axis, neuroinflammation, a*β*42, phospho-tau

## Abstract

Alzheimer’s disease (AD) is a progressive neurodegenerative disorder characterized by chronic neuroinflammation, metabolic dysfunction, and impaired proteostasis. Emerging evidence suggests that the gut microbiome influences brain function through the gut–brain axis; however, the mechanisms by which this communication affects AD pathology remain poorly understood. In this study, we used humanized *Drosophila* AD models expressing human AD-related transgenes in glial cells to investigate how glial AD pathology influences the gut microbiome and how microbiome depletion affects behavior and disease progression. Our findings revealed significant alterations in the abundance of several gut microbial taxa, including *Lactobacillus*, *Acetobacter, Enterococcus*, *Enterobacter*, *Pseudomonas*, *Leuconostoc*, *Providencia*, and *Gluconobacter*, suggesting a potential interaction between the gut microbiome and AD progression. To further define the role of the gut microbiome in AD progression, we treated *Drosophila* with antibiotics to achieve substantial microbiome depletion (SMD) and evaluated the resulting behavioral, physiological, and molecular changes. Compared with conventional controls, flies with SMD exhibited enhanced inflammatory signaling and cell death, impaired autophagy, lipid dysregulation, and increased accumulation of amyloid *β*-42 (a*β*42) and phospho-tau (pTau), with the extent of these changes varying among the different humanized AD models. These molecular alterations further exacerbated sleepcircadian disruption, altered locomotor performance, and worsened learning and memory deficits. Furthermore, we demonstrated a link between enhanced neuroinflammation and the accumulation of a*β*42, pTau, and amyloid precursor protein (APP) proteins. Collectively, our findings demonstrate that gut microbiome depletion aggravates glial-mediated AD pathology through the coordinated disruption of inflammatory, metabolic, and proteostatic pathways, ultimately leading to behavioral dysfunction. These results identify the gut microbiome as a critical regulator of glial homeostasis and provide new insights into its contribution to the progression of neurodegenerative disorders.

## Graphical abstract

**Figure:**
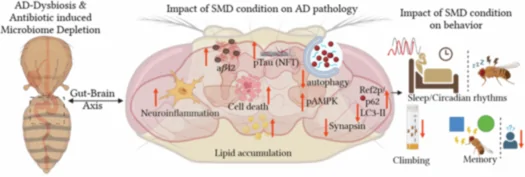

Illustration depicting how AD-associated dysbiosis and substantial microbiome depletion (SMD) affect AD pathology, including neuroinflammation, lipid accumulation, amyloid-*β* and phosphorylated tau accumulation, impaired autophagy, and cell death. The figure also illustrates the effects of AD pathology and SMD on synaptic integrity (Synapsin staining), cellular metabolism (pAMPK), and autophagy (Ref(2)P/p62 and LC3-II), as well as their associated behavioral outcomes, including alterations in sleep-circadian activity, climbing ability, and memory performance.

## 1. Introduction

Alzheimer’s disease (AD) is a progressive neurological disorder and a leading cause of dementia in older adults,^1^ affecting nearly 55 million people worldwide.^2^ AD is characterized by the progressive accumulation of amyloid beta (a*β*) plaques^3^ and hyperphosphorylated microtubule-associated protein tau, which forms neurofibrillary tangles (NFT).^4^ ^,^ ^5^ These protein aggregates could contribute to synaptic dysfunction, neurodegeneration, and cognitive decline. Although these pathological protein aggregates are considered hallmarks of AD, emerging evidence suggests that chronic neuroinflammation also contributes to disease progression.^6^ Activated glial cells, such as microglia and astrocytes, release proinflammatory cytokines that exacerbate neuronal dysfunction, disrupt synaptic homeostasis^7,^ and compromise protein clearance.^8^ ^,^ ^9^ Consequently, AD is now recognized as a multifactorial disorder in which interactions between neuroinflammation, metabolic dysfunction, and protein aggregation collectively contribute to progressive cognitive and behavioral impairments.^10^ ^,^ ^11^

Among the systemic factors contributing to AD pathology, the gut microbiome has emerged as a major contributing factor influencing host physiology through modulating metabolism, immune function, and gut–brain bidirectional communication. Several studies have reported an association between gut microbial dysbiosis and disease progression in patients with AD and animal models of the disease.^12‐14^ The microbiome can influence the central and peripheral immune response through microbial metabolites and cellular structural components^15‐19^ and can contribute to altered protein homeostasis and neuronal dysfunction. Although several studies support the functional link between the gut microbiome and AD,^20‐23^ the mechanisms by which the microbiome influences disease progression remain poorly understood. Specifically, the role of the gut microbiome in glial-mediated AD pathology has received limited attention. Even though neuroinflammation, protein aggregation, metabolic dysfunction, and synaptic impairment are well-defined characteristics of AD, it is still unclear whether alterations in the gut microbiome contribute to these pathological processes through glial-specific mechanisms. Understanding how gut microbial perturbations influence disease progression could provide new insights into microbiome-targeted therapeutic strategies for AD.

In this study, we investigated the role of the gut microbiome in modulating glial-mediated AD pathology using genetically tractable *Drosophila melanogaster* models expressing human AD-related transgenes in glial cells. By comparing conventional control (CC) and substantial microbiome depletion (SMD) conditions, we examined how microbiome depletion influences gut microbial composition, neuroinflammatory signaling, proteostasis, lipid homeostasis, and behavioral phenotypes. We hypothesized that microbiome depletion would differentially modify AD-associated molecular, cellular, and behavioral outcomes across transgenic models. Our findings reveal that the gut microbiome modulates glial-mediated AD pathology, with its depletion exacerbating neuroinflammation, protein accumulation, metabolic dysfunction, and behavioral impairments. These results provide insights into the contribution of gut–brain interactions to AD pathogenesis and highlight the gut microbiome as a potential regulator of glial homeostasis and neurodegenerative disease progression.

## 2. Materials and methods

### 2.1. *Drosophila* strains, generation of glial AD models and rearing conditions

The control and experimental flies were maintained in a standard diet containing active dry yeast 3 g/L, agar 11 g/L, yellow cornmeal 55 g/L, molasses 72 mL/L, propionic acid 6 mL/L, and 10% nipagin 8 mL/L. Flies were housed at 25 °C and 50% humidity under a 12-hour light-12-hour dark (LD) cycle. Fly food was changed every 3–4 d.^24-27^ Fly lines were obtained from the Bloomington *Drosophila* Stock Center (BDSC), and the GAL4/UAS system was used for cell and tissue-specific gene expression. For glial-specific expression, we used the *Glaz-GAL4* driver (BL#23353). Throughout the paper, (+) refers to *w* ^*1118*^ (BL#5905), UAS-GFP (BL#5431), human AD transgenic lines, such as UAS-APP (amyloid precursor protein, BL#64384), UAS-a*β*42 (BL#33769), and UAS-Tau (BL#51362) obtained from BDSC. The UAS-phospho Tau (BL#51365), UAS-Tau^*R406W*^ (obtained from Mel B Feany’s lab, Harvard Medical School), and UAS-Upd3 (obtained from Dr. Harrison's Laboratory, University of Kentucky, Lexington, KY, USA). The driver line was crossed with control and transgenic lines to obtain progeny expressing AD-associated transgenes specifically in glial cells. F1 progeny were collected at eclosion, separated by sex, and maintained for 3 weeks in separate vials containing 25 flies each.

### 2.2. Preparation of flies with substantial microbiome depletion

Parental flies were transferred onto fresh food daily to collect 24-hour-old embryos using embryo collection chambers (Flystuff® *Drosophila* Embryo Collection Cages-Genesee Scientific). 70% ethanol wash for 3–5 min,^28^ followed by rinsing with sterile water used to dechorionate embryos and to remove the external microbiome. These embryos were then transferred to standard fresh food and an antibiotic cocktail containing food vials. Flies were collected after eclosion and maintained for 3 weeks with food changed every 3 days^28^. Our lab has standardized microbiome depletion methods by changing standard fly food every 24 h to make microbiome-free flies along with antibiotic food, where we observed similar results.^28^ Based on the standardized protocol,^28-30^ Ampicillin (CAS No. 69-52-3), Kanamycin (CAS No. 25389-94-0), Tetracycline (CAS No. 60-54-8), Erythromycin (CAS No. 114-07-8), each one at a final concentration of 50  mg/L, were added to standard fly food after autoclaving at 60 °C to make antibiotic food. Flies that grow on standard food are termed as conventional control (CC), and on antibiotic food are termed as Substantial Microbiome Depletion (SMD), throughout the manuscript. The antibiotic treatment substantially reduces the gut microbial load,^28^ ^,^ ^30^ which we verified by universal 16S rRNA-specific primers to quantify microbiome abundance in head and body tissues using qPCR.

### 2.3. Experimental design and evaluation of comparisons

This study independently evaluated the impact of AD transgenes (Tau, Tau^*R406W*^, pTau, a*β*42, APP) expressed in glial cells and how microbiome depletion influences molecular, behavioral, and pathological phenotypes. We included three predefined comparisons throughout the study. First, we compared AD transgenic flies with control (Glaz/+) and GFP flies under CC conditions to characterize disease-associated phenotypes in the presence of a conventional microbiome. Second, we compared the same groups under SMD conditions to demonstratedisease-associated phenotypes following microbiome depletion. Finally, we compared CC and SMD flies within each genotype to assess the effects of antibiotic-associated microbiome depletion. All behavioral, physiological, and molecular analyzes were performed using coded sample names to minimize experimental bias. Further to complement statistical significance, the “effect size comparisons” (Cohen’s d) were calculated for all matched genotypes in CC and SMD conditions to quantify the magnitude of microbiome-dependent effects. For each figure, Cohen’s d values are calculated and provided in the source data file.

### 2.4. Sleep/circadian activity

A *Drosophila* activity monitor system (DAM, TriKinetics, Inc., MA, USA) was used to measure fly sleep/circadian activity behavior under a 12-hour light:12-hour dark cycle at 25 °C for 5 consecutive days.^31^ ^,^ ^32^ Male flies (2.5-week-old) were housed in a glass tube containing either standard or antibiotic-supplemented food, depending on the experimental condition. Three biological replicates (*n* = 3) were used, with 16 flies in each replicate (48 flies per genotype per condition). We measured day, night, and total sleep/activity (wake-up) duration during 24 h for 5 consecutive days. Raw data were used to quantify sleep (immobility of a fly > 5 min), activity (fly crossing the infrared beam at the center of the DAM system), and sleep bout (inactiveness of fly for a minimum of 5 consecutive minutes) and bout length (duration of each sleep period/sleep bout).^33^ We quantified the sleep fragmentation index by dividing the sleep bouts by bout length, which provides details about individual fly sleep quality. The average sleep/activity behavior of individual flies from each genotype and condition in 24 h (ZT0-ZT24) was calculated using in-house “One-Click sleep analysis software application” developed in the Python background.^34^

### 2.5. Assessment of locomotory performance using geotaxis assay

Locomotion/climbing behavior was assessed using a 3D-printed motor-mounted automatic device (designed and developed based on a Python script)^35^ to test the locomotory performance of our experimental flies. The device was equipped with a motor arm, tube holder, Raspberry Pi camera connected to a 4B monitor, and a light source to clearly capture, observe, and record the fly's movements while performing the assay. We used 11 flies per biological replicate (*n* = 3). Videos were analyzed using a custom Python-based automated system incorporating computer vision and object detection algorithms to quantify the mean climbing performance across biological replicates. The final output files contain relevant plots and .csv files with fly performance data during the assay.

### 2.6. Olfactory aversive training and memory assessment in *Drosophila*

T-maze instrument (CelExplorer Labs) provided with two neutral odors (4-methylcyclohexanol and 3-octanol) diluted in mineral oil (1:10 ratio), coupled with an electric shock device, was used to assess the memory behavior of flies. The flies were grouped into 30‒40 per experiment from each genotype per biological replicate (*n* = 3), and the assay was performed based on a standard protocol.^36^ The assay consists of three phases (Naive-*N*, Training-T, and Testing). In Naïve-phase flies were allowed to explore odor choices in the T-maze for 3.5 min to determine the odor preferences of flies and score odor choices. During the training phase, the flies were exposed to electric shock (100–110 volts) followed by non-shock sessions for 2.5 min in each session based on the most preferred odor of flies during Naïve phase of the experiment. The training phase was repeated three times, followed by a 5 min resting period. In the third phase of the experiment, flies were exposed to two odors simultaneously for 3.5 min, and then the chambers were closed to retain the flies in the respective odor chambers to score the flies. Based on fly preference for odor, memory performance was assessed and represented as a performance index (calculated by counting the number of flies choosing shock odor, subtracting with flies choosing non-shock odor and divided with total number of flies).^37^ ^,^ ^38^ The avoidance index (calculated by dividing the number of flies choosing non-shock odor by the total number of experimental flies, then multiplying by 100).^38^

### 2.7. Cytological analysis

Flies were dissected and immediately transferred into 4%PFA in phosphate-buffered saline (PBS) for 15 min with mild agitation at room temperature. PFA was removed by washing with 1× PBS (3 × 10 min each) and transferred into 10% sucrose in PBS solution overnight. Then tissues were embedded in OCT (Fisher Health care#4585) medium for cryosectioning, and 20 µm thin sections were prepared and transferred onto silicon-coated slides (Globe scientific#1358A).^26^ ^,^ ^39^ The tissues were stained with primary antibodies (Synapsin, 1:250 UI Developmental Studies Hybridoma Bank #3C11), phalloidin (Abnova U0281), APP (IMG-190-2, IMAGENEX), and a*β*42 (6E10, Novus Bio) overnight and then washed. Secondary antibodies, Alexa Fluor 750 anti-mouse and Alexa Fluor 488 anti-rabbit 1:1500, were added to the slides and incubated for 1 h at room temperature (Thermo Fisher #A-21037). For lipid analysis, we used Lipid Spot-488, 1:100 dilution (Biotinum #70065). Finally, we washed the slides three times with 1× PBS for 5 min each. We used VECTASHEILD Vibrance H-1800, which contains antifade mounting medium with DAPI (0.9 µg/ml). The slides were left overnight in the dark, and then images were captured using multichannel fluorescence microscopy (Olympus BX63). The images were analyzed using CellSens software at 10x magnification.

The region of interest (ROI) was defined using the DAPI channel. It distinguished the anatomical subregions in the head and in the body. Two ROIs were drawn for the head and brain separately. One ROI was drawn for the body region. For each ROI, mean object area and object size were collected at the 488 nm channel, and mean pixel intensity was collected at the 750 nm channel. From each genotype, multiple sections were analyzed from head and body regions to measure the mean object count and mean object area. Lipid and APP channels (488 nm) were thresholded to minimize the background signal. The minimum detection size of an object is set to 20 pixels. Synapsin and a*β*42 (750 nm) channels were not thresholded to retain the integrity of the comparison between groups and conditions. Essential background filters and thresholding values were applied before taking the images for all images across groups. To reduce bias, we collected data from multiple sections per fly (*n* = 3) and multiple flies for each condition (*n* = 5), i.e., 15 sections/genotype for head and body regions separately in all groups. Watershed segmentation and Otsu thresholding were applied to all images for quantification.

### 2.8. Gene expression analysis

Dissected fly tissues were snap-frozen in liquid N_2_ and used for RNA isolation using Zymo Research Quick-RNA Micro Prep Kit (Catalog R1051, Zymo Research, Irvine, California, USA).^31^ ^,^ ^40^ A BioTek SYNERGY|LX multi-mode reader was used to check RNA purity and quantity. cDNA was prepared using BioRad iScript™ Reverse Transcription Supermix (Catalog #170-8840). Quantitative PCR was conducted using the Sso advanced universal SYBR Green supermix from Bio-Rad (Catalog #1725270) using the Bio-Rad CFX Opus Real-Time PCR system. No-reverse transcriptase (No-RT) controls and melt-curve analysis were used to confirm primer specificity and the absence of contamination. The results represent 2^−ΔΔCt^ values normalized to RpL11 gene expression and control samples. All the qPCRs were performed using biological triplicates (*n* = 3). The forward and reverse primers used for quantitative PCR are mentioned in the source data file (source data file Figure 3).

### 2.9. Microbiome analysis

The experimental flies were washed with 70% ethanol and dissected to remove wings and limbs. Head and body (thorax and abdomen) tissues were collected, and the microbial DNA was isolated using the DNeasy Ultraclean Microbial kit from Qiagen (catalog # 1222450).^28^ DNA was quantified using a BioTek SYNERGY|LX multi-mode reader. The DNA quantified and used was 5 ng of pure DNA for each qPCR reaction using genus- and species-specific primers listed in the source data file (Figure 1). Quantitative PCR was performed using the Sso advanced universal SYBR Green supermix from Bio-Rad (Catalog #1725270) using the Bio-Rad CFX Opus Real-Time PCR system. Further, we also included a negative control to measure environmental contamination. Results represent 2^−ΔΔCt^ values normalized with RpL11 gene expression and control samples. For each biological replicate, we used 8–10 flies, washing with Milli-Q water followed by removing the head and limbs to minimize external microbial contamination. All the qPCRs were performed using biological triplicates (*n* = 3). Primers used for qPCR analysis of microbiomes are mentioned in the source data file (Source data file Figure 1).

**Figure 1.**
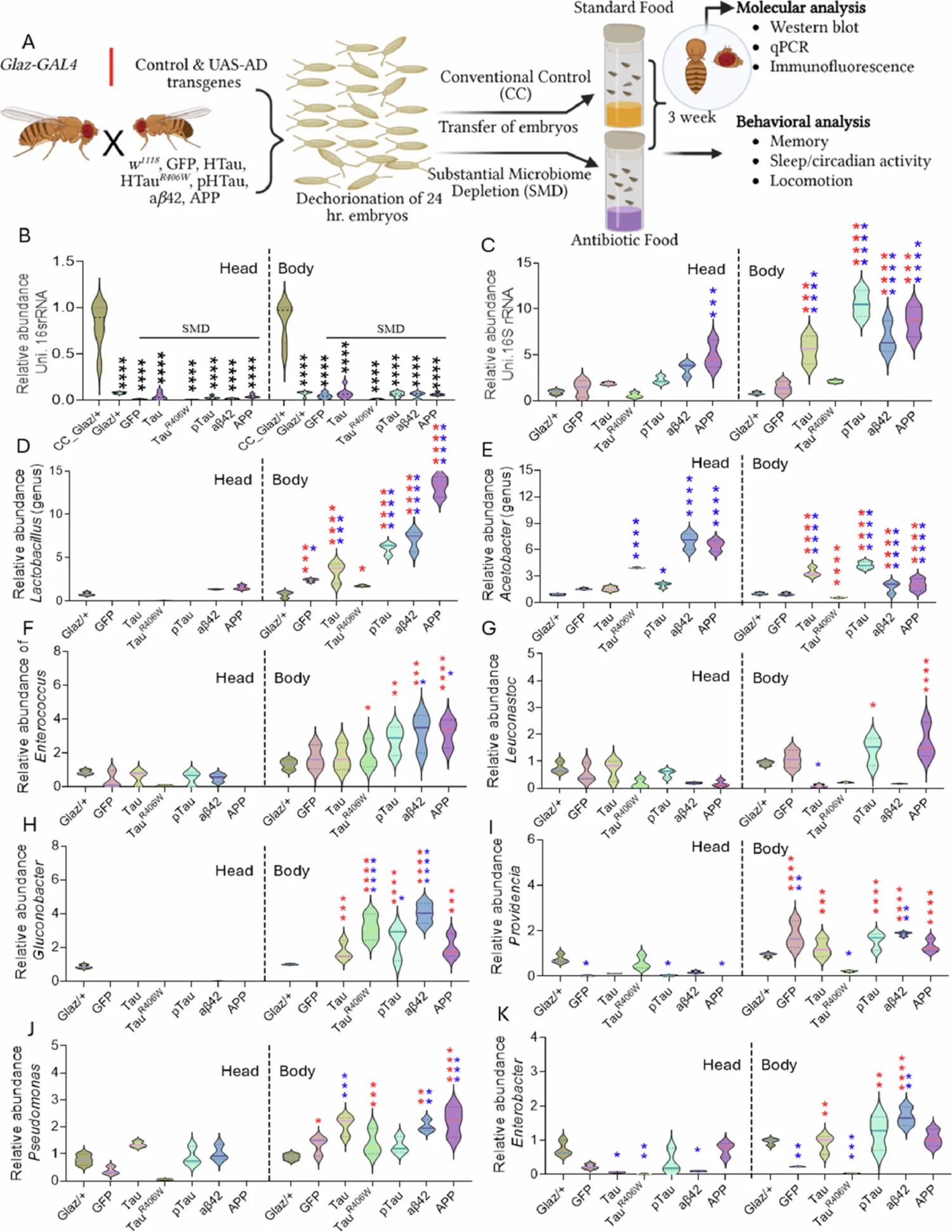
Experimental design and quantification of microbiome abundance in the head and body tissues of humanized AD *Drosophila* models. (A) Schematic illustrating the fly genotypes and experimental procedures used in this study, as detailed under the mathods-section. (B) Quantification of microbiome in substantially microbiome-depleted (SMD) compared toconventional control (CC) group in control and Alzheimer disease (AD) transgenic flies in head and body tissues using universal 16S rRNA gene-specific primers. (C) Quantification of total microbiome abundance in the head and body tissues of control and AD transgenic flies reared under CC conditions using universal 16S rRNA gene-specific primers. (D–K) Quantification of the relative abundance of individual bacterial genera in control and AD transgenic flies: *Lactobacillus* (D), *Acetobacter* (E), *Enterococcus* (F), *Leuconostoc* (G), *Gluconobacter* (H), *Providencia* (I), *Pseudomonas* (J), and *Enterobacter* (K). For panel B, one-way ANOVA followed by Dunnett’s multiple-comparisons test was used to compare CC controls with all SMD groups. For panels C–K, two-way ANOVA followed by Šídák’s multiple-comparisons test was performed. Blue asterisks indicate comparisons between *Glaz/+* controls and AD genotypes within each condition, whereas red asterisks indicate comparisons between identical genotypes under CC and SMD conditions. Data represent three biological replicates per genotype and condition, with each replicate comprising 8–10 flies. Statistical significance is indicated as follows: *P* < 0.05, *P* < 0.01, *P* < 0.001, and *P* < 0.0001. Raw data and exact *P* values are provided in the source data file.

### 2.10. Western blot analysis

Western blot analysis was performed using dissected experimental fly head tissues. Protein isolation was performed using RIPA buffer (Thermo Scientific, Pierce^TM^ RIPA buffer) provided with protease and phosphatase inhibitors (Thermo Scientific, Halt^TM^ catalog **#**78441) in a 1.5 ml Eppendorf tube. The heads were homogenized with a handheld homogenizer (Fisher brand motorized tissue grinder 12-1413-61) on ice, and tubes with sample lysate were centrifuged at 16,000  rpm for 15 min at 4 °C according to standard protocol.^41^ The supernatant was collected; protein quantity was estimated using Bradford reagent (BioRad catalog #5000006) and a BioTek SYNERGY multimode plate reader at 595 nm. 2x sample buffer (BioRad catalog #1610737) was added to protein samples and boiled at 100 °C for 5 min. Proteins were separated by running SDS–PAGE using precast mini gels (Bio-Rad catalog #4561036) and Bio-Rad tri-color protein marker (Bio-Rad catalog #1610374). The gels were subjected to overnight transfer at 4 °C at 25 volts using transfer buffer with 20% methanol onto a 0.2 µm nitrocellulose membrane (Bio-Rad catalog #1620112). The blots were blocked with 3% BSA (Fisher Scientific) for 1 h at room temperature and washed using TBS briefly. Primary antibodies Tau (DSHB, 5A6-C), pTau Ser 202, Thr205 (Invitrogen, MN1020), a*β*42 (6E10, Covance catalog # SIG-39320; Sigma Aldrich, AB1510), Synaptotagmin (DSHB3H2 2D7), Ref(2)p (Ab178440), ubiquitin (AbcamEP8589), AMPK (Ab80039), pAMPK (AbcamEPR5683), GABARAP (CST-E1J4E#13733), APP (IMG-190-2, IMAGENEX), Stat5A-7D8 (DSHB, AB2617948) and *α*-tubulin (DSHB4A1) in TBST and the blots were incubated overnight at 4 °C. Blots were washed with TBS-TBST-TBS for 30 min (10 min each), then added secondary anti-rabbit (Abcam #ab6721), or anti mouse (Abcam #ab6728) tagged with HRP and incubated at room temperature for 1 h. Excess secondary antibody was washed with TBST-TBS-TBST (10 min each) for 30 min. ECL reagents (Bio-Rad) were used to develop the blots using an Amersham 600 ChemiDoc with chemiluminometric reading. To confirm equal protein loading across the samples, normalization was performed using *α*-tubulin. Chemiluminescence exposures were kept within the linear detection range. Quantification of blots was performed using Image-J, and used similar quantification parameters across all experimental groups. 8–10 heads and body tissues were used in each replicate (*n* = 3).

## 3. Statistical analysis

Statistical analyses were carried out using GraphPad Prism version 10. Each AD transgenic genotype was compared with the control (*Glaz*/+) and *Glaz > GFP* (provided in supplementary file) in each group and between matched genotypes in CC and SMD conditions. Data are presented as mean ± SD. Statistical significance was indicated as follows: *p* < 0.05 (*), *p* < 0.01 (**), *p* < 0.001 (***), and *p* < 0.0001 (****). An outlier test is performed using the modified Z-score method.^42^ Detailed statistical analyses among genotypes and between CC and SMD conditions are provided in the source data file. Moreover, as indicated under each figure legend, we used one-way ANOVA followed by Dunnett’s post hoc test, and Two-way ANOVA followed by Šídák’s multiple comparisons test for microbiome analysis. Two-way ANOVA followed by Šídák’s multiple comparisons test was used for immunofluorescence, qPCR, western blot analysis, memory assay, and one-way ANOVA followed by Šídák’s multiple comparisons test for geotaxis analysis. The chi-square test was also used for the memory assay. Further to complement the *p*-values. The “effect size comparisons” (Cohen’s d) were calculated for all matched genotypes between CC and SMD conditions to quantify the magnitude of microbiome-dependent effects. Cohen’s d-values: small effect: d = 0.2, medium effect: d = 0.5, large effect: d = 0.8, very large effect: d = 1.2, and huge effect: d = 2.0 (negative d-values represent a significant increase and positive d-values represent a significant reduction). Specific sample sizes, biological replicates, and statistical tests used were provided in the corresponding figure legends, and adjusted *p*-values and Cohen’s d values were provided in the source data file.

### 3.1. Results

#### 3.1.1. Humanized AD flies exhibit genotype-specific alterations in bacterial abundance, and antibiotic treatment substantially reduces bacterial load

To examine how glial AD pathology affects bacterial abundance, we established conventional control (CC) and antibiotic-treated, substantially microbiome-depleted (SMD) groups. Figure 1A outlines the experimental procedures. Figure 1B shows relative bacterial abundance in SMD flies compared to Glaz/+ in CC flies, with universal 16S rRNA-specific primers using qPCR. Flies reared in antibiotic conditions exhibited ~90% reduction in bacterial abundance (Figure 1B, S.I. Figure 1). We next quantified bacterial abundance using universal 16S rRNA-specific primers. We found a significant bacterial abundance in APP flies heads (*p* < 0.003) and increased bacterial abundance in the body region of Tau, pTau, a*β*42, and APP (adjusted *p* < 0.0001) flies compared to respective controls. These results indicate differences in bacterial abundance between head and body tissues (Figure 1C).

To determine whether these changes extended beyond overall bacterial abundance, we quantified the most abundant bacterial genera in the fly gut, i.e., *Lactobacillus* and *Acetobacter* ^28^ ^,^ ^43^ ^,^ ^44^ in our AD *Drosophila* models using qPCR. Bacteria belonging to the *Lactobacillus* genus (Figure 1D) showed a significant increase in GFP (*p* < 0.04), Tau (*p* < 0.0005), pTau, a*β*42, and APP flies (*p* < 0.0001) compared to the control in the body region. Compared to the head, the body region showed a significant increase in microbiome abundance (adjusted *p* < 0.0001). In the body region, *L. plantarum* (S.I. Figure 1I) showed a significant increase in Tau, Tau^*R406W*^, pTau, a*β*42, and APP (*p* < 0.0001) in flies compared to the control and head region of corresponding flies (adjusted *p* < 0.0001). *L. brevis* (S.I. Figure 1J) showed no significant increase in the body of Tau, pTau, and APP flies (adjusted *p* < 0.0001) compared to control. *Acetobacter* (Figure 1E) showed an increase in Tau^*R406W*^, a*β*42, APP (*p* < 0.0001), and pTau (*p* < 0.03) compared to control in the head region. In the body region, Tau, pTau, a*β*42, and APP (*p* < 0.0001) showed increased abundance compared to control and compared to corresponding head regions. *A. promorum* (S.I. Figure 1 K) showed a significant increase in Tau, Tau^*R406W*^, pTau, a*β*42, and APP (adjusted *p* < 0.001) compared to control in the body region and compared to the corresponding head regions. *A. pasteurianus* (S.I. Figure 1 L) showed a significant increase in the APP head region (*p* < 0.0001), compared to control and Tau, pTau, a*β*42, and APP (adjusted *p* < 0.001) in the body region, compared to control and the head region of corresponding flies. Based on our study, we found significant differences in the regional distribution of *Lactobacillus* and *Acetobacter* and associated taxa (Figure 1D, E and S.I. Figure 1I–L).

We also quantified additional bacterial genera to examine whether AD-related transgene expression was associated with changes beyond the dominant taxa. These included *Enterococcus, Gluconobacter, Providencia, Enterobacter*, *Leuconostoc*, and *Pseudomonas*. These bacterial genera showed low or undetectable abundance in head tissues, whereas significant differences were observed only in body tissues. *Enterococcus* (Figure 1F) showed a significant increase in a*β*42 and APP (*p* < 0.02) in flies compared with controls. No significant increase in *Leuconostoc* (Figure 1G) was observed. *Gluconobacter* (Figure 1H) showed increased abundance in Tau^*R406W*^, pTau (*p* < 0.0001), and a*β*42 (*p* < 0.03) compared to control. *Providencia* (Figure 1I) abundance was found to be significant in GFP and a*β*42 (*p*, 0.004) compared to control. *Pseudomonas* (Figure 1J) abundance showed a significant increase in Tau, APP (*p* < 0.0006), and a*β*42 (*p*,0.007) compared to control. *Enterobacter* (Figure 1K) abundance showed an increase in a*β*42 (*p*, 0.004) with no increase in other genotypes. In addition, we also performed genotype-specific comparisons for major bacteria using genus- and species-specific primers (S.I. Figure 1 A–H). Our findings showed differences in the abundance of both dominant and less abundant bacterial taxa across glial AD *Drosophila* models under conventional conditions.

#### 3.1.2. Microbiome depletion alters lipid homeostasis, acto-myosin network, and synaptic protein abundance in glial AD models

To determine whether microbiomes influence lipid homeostasis and synaptic protein abundance, and the acto-myosin network during glial-mediated AD pathology. We checked the head, brain, and abdomen regions of control and AD transgenic flies for lipid accumulation. We compared control and AD transgenic flies within each condition, and also performed genotype-matched comparisons between CC and SMD to assess the effects of microbiome depletion. Effect size comparisons (Cohen’s d) for all matched CC and SMD fly genotypes were calculated and represented in the source data file for Figure 2. The lipid object counts in the head (Figure 2B) were found to be significantly increased in Tau^*R406W*^ (*p* < 0.0001) and pTau (*p* < 0.012) compared to SMD control (*Glaz/+*) and CC (*p* < 0.0001) counterpart flies. Effect size comparison between CC and SMD flies suggests a significant increase in lipid object count in SMD flies: Glaz/+(*d* = −0.69), GFP (*d* = −1.29), Tau (*d* = −1.48), Tau^*R406W*^ (*d* = −1.73), pTau (*d* = −2.18), a*β*42 (*d* = −0.43), and APP (*d* = −0.28) compared to respective genotypes in CC condition. Lipid object area in the head (Figure 2C) showed A significant reduction in Tau (*p* < 0.0075), GFP, Tau^*R406W*^, pTau, a*β*42, and APP (*p* < 0.0001) in SMD flies compared to control and decreased even with CC GFP and a*β*42 flies. Effect size comparison also suggests a significant reduction in lipid object area in the head of SMD flies GFP (*d* = 1.60), Tau (*d* = 0.15), Tau^*R406W*^ (*d* = 0.40), pTau (*d* = 1.56), a*β*42 (*d* = 1.53), and APP (*d* = −0.58) compared with CC flies, except Glaz/+(*d* = −1.68). In the brain region, lipid object count (Figure 2D) was increased in APP flies (*p* < 0.0001) compared to control in CC, and Tau (*p* < 0.0001) and pTau (*p* < 0.01) in SMD flies. We also observed a significant reduction in APP flies (*p* < 0.0001) in the SMD condition compared to CC flies. Effect size comparison between CC and SMD flies suggests lipid object counts in the brain of SMD flies showed an increase in Glaz/+(*d* = −0.58), GFP (*d* = −1.26), Tau (*d* = −0.75), Tau^*R406W*^ (*d* = −0.65), pTau (*d* = −1.59), and a decrease in a*β*42 (*d* = 0.57) and APP (*d* = 3.09) compared to CC flies. Lipid object area in the brain (Figure 2E) was reduced compared to SMD control in pTau (*p* < 0.001), Tau^*R406W*^, a*β*42, and in APP (*p* < 0.0001), but the effect size comparison for lipid object area in the brain significantly increased in Glaz/+ (*d* = −2.11), GFP (*d* = −1.23), Tau (*d* = −0.34), and pTau (*d* = −0.57) compared CC flies.

**Figure 2.**
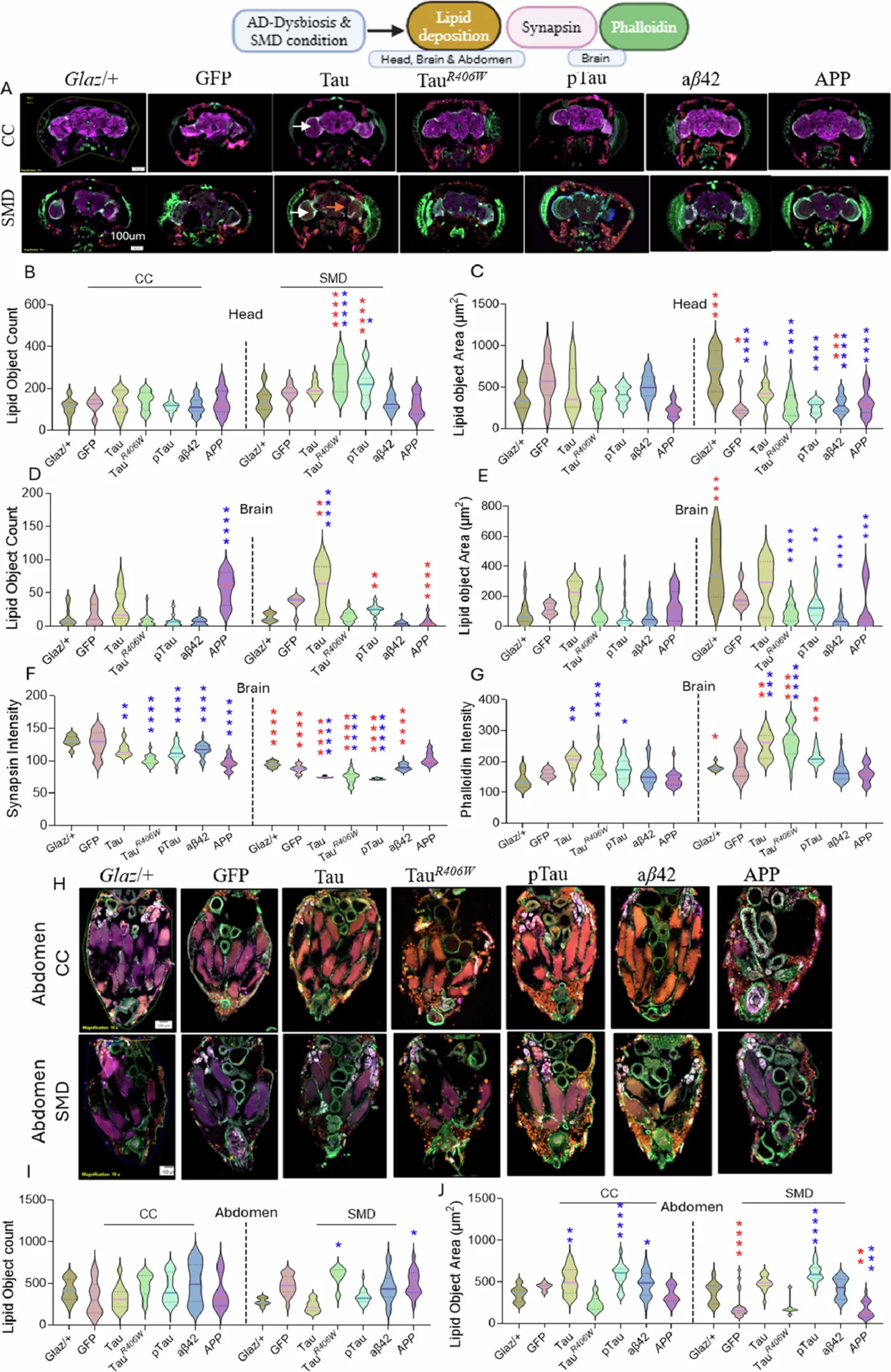
Microbiome depletion alters lipid accumulation, acto-myosin network and synaptic protein levels in humanized ADmodels. Representative fluorescence images show lipid droplets (orange), Synapsin (magenta), and phalloidin (green). (A–G) Analysis of head and brain tissues: (A) Representative staining of fly heads; (B, C) quantification of lipid droplet number and area in the head, respectively; (D, E) quantification of lipid droplet number and area in the brain, respectively; (F) quantification of Synapsin fluorescence intensity in the brain; and (G) quantification of phalloidin fluorescence intensity in the brain. (H–J) Analysis of abdominal tissues: (H) Representative fluorescence images of the abdomen; (I, J) quantification of lipid droplet number and area in the abdomen, respectively. Blue asterisks indicate comparisons between *Glaz/+* controls and AD genotypes within each microbiome condition, whereas red asterisks indicate comparisons between identical genotypes maintained under conventional control (CC) and substantial microbiome depletion (SMD) conditions. Statistical significance was assessed using two-way ANOVA followed by Šídák’s multiple-comparisons test. Data represent *n* = 5 per genotype and condition, with three tissue sections analyzed per fly. Statistical significance is indicated as follows: *P* < 0.05, *P* < 0.01, *P* < 0.001, and *P* < 0.0001. Raw data and exact *P* values are provided in the source data file.

To determine whether altered lipid homeostasis was accompanied by changes in synaptic protein abundance and acto-myosin network, we next quantified synapsin (Figure 2F) and phalloidin (Figure 2G) staining. We found a significant reduction in synapsin intensity (Figure 2F) in the brain region of Tau (*p* < 0.01), Tau^*R406W*^, pTau, a*β*42, and in APP flies in the CC and all the flies in the SMD condition (*p* < 0.0001) compared to CC control. Whereas in SMD flies, Tau, Tau^*R406W*^, pTau showed (*p* < 0.0001) further reduction in synapsin intensity compared to SMD control flies. The effect size comparison suggests synapsin intensity in the brain was significantly reduced in Glaz/+ (*d* = 4.75), GFP (*d* = 3.14), Tau (*d* = 5.67), Tau^*R406W*^ (*d* = 3.12), pTau (*d* = 5.30), and a*β*42 (*d* = 2.63) compared to CC flies. Phalloidin staining (Figure 2G) showed a significant increase in Tau (*p* < 0.0014), Tau^*R406W*^ (*p* < 0.0003), and pTau (*p* < 0.031) in CC, and Tau (*p* < 0.0005) and Tau^*R406W*^ (*p* < 0.0001) in SMD flies compared to control flies. In SMD flies, we further observed an increase in phalloidin staining in *Glaz/+* (*p* < 0.03), Tau (*p* < 0.002), Tau^*R406W*^ (*p* < 0.0003), and pTau (*p* < 0.027) compared to CC flies. The effect size comparison suggests that phalloidin intensity in brain was significantly increased in Glaz/+ (*d* = −1.78), GFP (*d* = −0.81), Tau (*d* = −1.50), Tau^*R406W*^ (*d* = −1.67), pTau (*d* = −1.41), a*β*42 (*d* = −0.34), and APP (*d* = −0.75) compared to CC flies.

In the abdomen region, lipid object count (Figure 2I) in Tau^*R406W*^ (*p* < 0.016) and APP flies (*p* < 0.046) in the SMD condition showed a significant increase compared to control. Further effect size comparison between CC and SMD flies suggests that lipid object area in the abdomen showed significant changes in Glaz/+ (*d* = −0.15), GFP (*d* = 1.67), Tau (*d* = 0.22), Tau^*R406W*^ (*d* = 0.42), pTau (*d* = −0.23), a*β*42 (*d* = 0.33), and APP (*d* = 1.68) compared CC flies. The lipid object area in the abdomen (Figure 2 J) showed a significant increase in Tau (*p* < 0.003), pTau (*p* < 0.0001), and a*β*42 (*p* < 0.034) in CC flies compared to control. Whereas in SMD there is a significant increase in pTau (*p* < 0.0001) and a decrease in APP (*p* < 0.001) compared to SMD control. GFP flies showed a significant reduction compared with CC and SMD controls (*p* < 0.0001). The effect size reporting suggests lipid droplet area in the abdomen showed change in Glaz/+ (*d* = 1.26), GFP (*d* = −0.98), Tau (*d* = 0.88), Tau^*R406W*^ (*d* = −0.78), pTau (*d* = 0.73), a*β*42 (*d* = 0.23), and APP (*d* = −0.63) compared with CC flies. These results showed genotype-dependent differences in lipid droplet counts and area between CC and SMD flies across head, brain, and abdominal tissues.

#### 3.1.3. Microbiome depletion increases the expression of inflammation-related genes in glial AD models

To examine how the microbiome influences inflammation-associated gene expression during glial-mediated AD pathology, we quantified the mRNA expression of cytokines (*Upd1-3*), components of the JAK/STAT pathway (*Dome, Hop,* and *Stat92e*), the TNF homolog *Eiger*, and its downstream effector *Imd*. We also measured the apoptosis-related genes *Hid* and *Reaper* in the head and body regions of CC and SMD, AD flies, along with controls. We compared control and AD transgenic flies within each condition and performed genotype-matched CC and SMD comparisons to assess changes in gene expression associated with microbiome depletion. Effect size comparisons (Cohen’s d) for all matched CC and SMD fly genotypes were calculated and represented in the source data file for Figure 3 and S.I. Figure 3. In the heads of *Upd3* (*IL6*), it was found to be decreased (Figure 3A) in GFP (*p* < 0.03) and increased in APP (*p* < 0.03) compared to control in the CC condition. In SMD condition, Tau^*R406W*^ (*p* < 0.0003), a*β*42 and APP (*p* < 0.0001) flies showed a significant increase compared to control and a significant increase in Tau^*R406W*^, a*β*42 (*p* < 0.0001) in SMD compared to CC flies. The effect size comparison between CC and SMD flies suggests a significant increase in *Upd3* mRNA expression in the head of Glaz/+ (*d* = −0.41), GFP (*d* = −0.11), Tau (*d* = −1.45), Tau^*R406W*^ (*d* = −4.27), pTau (*d* = −0.52), a*β*42 (*d* = −4.14), and APP (*d* = −2.21) compared to respective genotypes in the CC condition. The UPD3 cytokine receptor gene *Dome* (Figure 3B) showed a significant increase in a*β*42 (*p* < 0.0035) and APP (*p* < 0.04) in CC flies compared to controls. In SMD flies, Tau^*R406W*^ (*p* < 0.0018), a*β*42, and APP flies (*p* < 0.0001) showed a significant increase compared to SMD control flies. Compared to CC, Tau^*R406W*^ (*p* < 0.0032), a*β*42, and APP (*p* < 0.0001) flies showed a significant increase, and pTau (*p* < 0.048) showed a significant decrease in the SMD condition. The effect size comparison suggests that *Dome* mRNA expression showed a significant increase in SMD head of Glaz/+ (*d* = −0.48), GFP (*d* = −3.40), Tau (*d* = −2.65), Tau^*R406W*^ (*d* = −5.21), pTau (*d* = −3.86), a*β*42 (*d* = −3.75), and APP (*d* = −3.03) compared with CC flies. The cytoplasmic downstream target of DOME is known as *Hop* (Figure 3C) showed significant increase in a*β*42 (*p* < 0.0024) and APP (*p* < 0.0001) in CC compared to controls. In SMD condition Tau^*R406W*^, a*β*42 and APP (*p* < 0.0001) flies showed a significant increase compared to SMD control flies. Further, compared to CC flies, SMD Tau^*R406W*^ (*p* < 0.00012), a*β*42 (*p* < 0.0028), and APP flies (*p* < 0.0001) showed a significant increase in *Hop*. The effect size comparison of *Hop* mRNA expression between CC and SMD suggests a significant increase in the head of Glaz/+ in SMD flies (*d* = −1.34), GFP (*d* = −2.56), Tau (*d* = −1.09), Tau^*R406W*^ (*d* = −7.73), pTau (*d* = −1.15), a*β*42 (*d* = −5.29), and APP (*d* = −3.33) compared with CC flies. In the SMD condition, Tau^*R406W*^ (*p* < 0.004), pTau (*p* < 0.0003), and APP flies (*p* < 0.0004) showed significant upregulation of *Stat92e* compared to SMD control flies (Figure 3D). Further, compared to CC flies, SMD, Tau^*R406W*^ (*p* < 0.001), a*β*42 (*p* < 0.003), and APP flies (*p* < 0.014) showed a significant increase in *Stat92e* gene expression. The effect size comparison between CC and SMD flies suggests a significant increase in *Stat92e* mRNA in the head of Glaz/+  in SMD flies (*d* = −0.40), GFP (*d* = −2.79), Tau (*d* = −3.54), Tau^*R406W*^ (*d* = −3.94), pTau (*d* = −1.65), a*β*42 (*d* = −2.30), and APP (*d* = −2.40) compared with CC flies.

**Figure 3.**
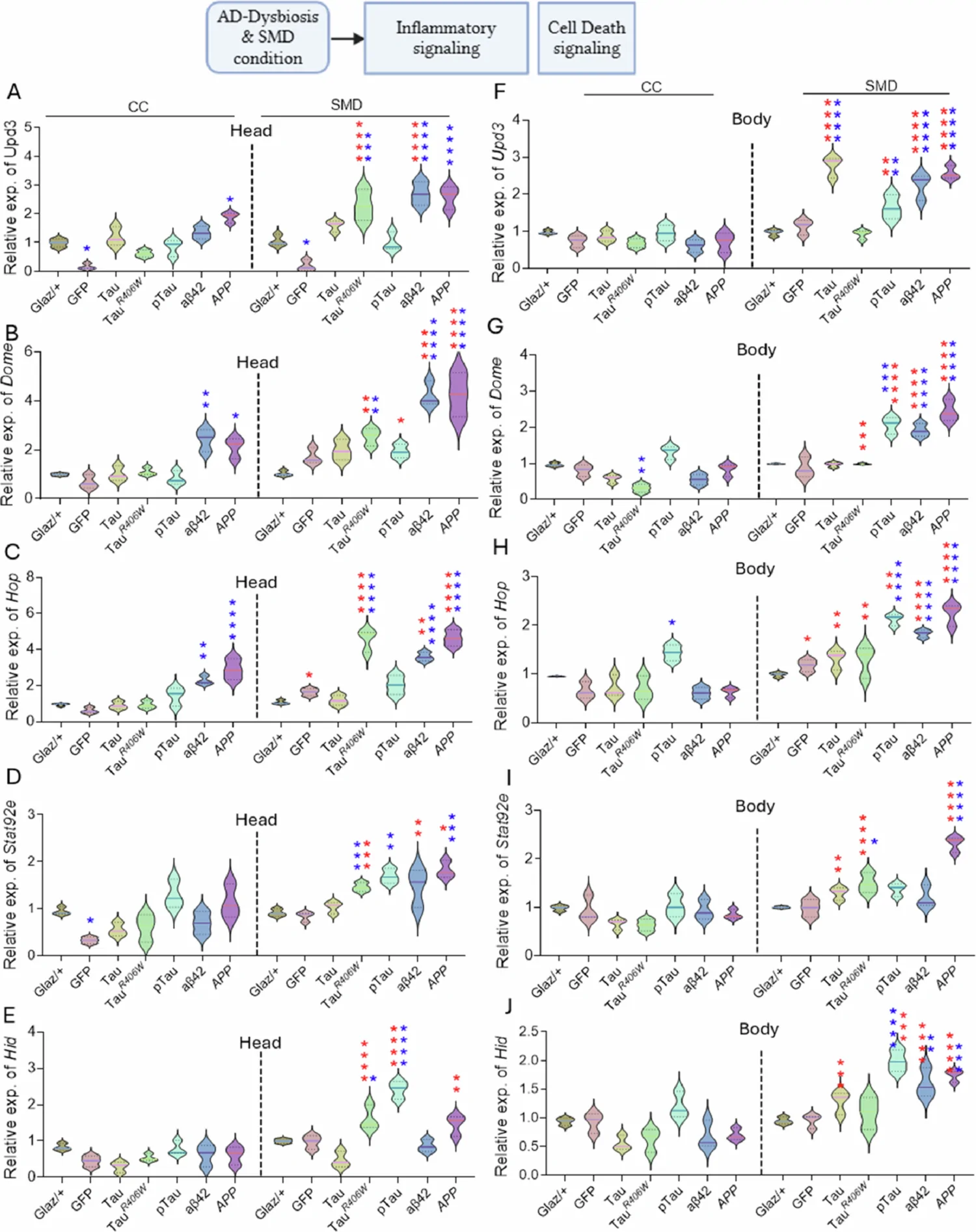
Microbiome depletion exacerbates AD-associated inflammatory signaling and cell death marker expression in the head and body of humanized AD *Drosophila* models. Quantitative PCR (qPCR) analysis of inflammatory gene expression in 3-week-old flies maintained under conventional control (CC) or substantial microbiome depletion (SMD) conditions. (A–E) Gene expression analysis in head tissues: (A) *Upd3*, (B) *Dome*, (C) *Hop*, (D) *Stat92E*, and (E) the cell death-associated marker *Hid*. (F–J) Gene expression analysis in body tissues: (F) *Upd3*, (G) *Dome*, (H) *Hop*, (I) *Stat92E*, and (J) *Hid*. Blue asterisks indicate comparisons between *Glaz/+* controls and AD genotypes within each microbiome condition, whereas red asterisks indicate comparisons between identical genotypes maintained under CC and SMD conditions. Statistical significance was assessed using two-way ANOVA followed by Šídák’s multiple-comparisons test to evaluate genotype and microbiome condition effects. Data represent three biological replicates per genotype and condition, with each replicate comprising 8–10 flies. Statistical significance is indicated as follows: *P* < 0.05, *P* < 0.01, *P* < 0.001, and *P* < 0.0001. Raw data and exact *P* values are provided in the source data file.

We then checked the possible implication of inflammation in promoting cell death. We have checked *Hid* (Figure 3E), which is an inhibitor of *Drosophila* apoptosis inhibitory protein1 (DIAP1).^45^ In our study, we found a significant increase in Tau^*R406W*^ (*p* < 0.03) and pTau (*p* < 0.0001) in SMD flies compared to SMD control flies. Further, compared to CC flies, Tau^*R406W*^ (*p* < 0.0001), pTau (*p* < 0.0001), and APP flies (*p* < 0.0009) showed a significant increase in *Hid* expression in the SMD condition. In CC flies, we did not observe any significant change in *Hid* expression compared to control. The effect size comparison suggests a significant increase in *Hid* mRNA expression in Glaz/+ head of SMD flies (*d* = −2.04), GFP (*d* = −3.06), Tau (*d* = −0.95), Tau^*R406W*^ (*d* = −4.25), pTau (*d* = −7.27), a*β*42 (*d* = −1.02), and APP (*d* = −3.12) compared with CC flies.

We further checked *Upd*3 (Figure 3F), *Dome* (Figure 3G)*, Hop* (Figure 3H)*, Stat92e* (Figure 3I), and *Hid* (Figure 3J) in the abdominal region to understand the influence of the gut microbiome on peripheral inflammatory responses. In the SMD condition (Figure 3F), Tau, a*β*42, APP (*p* < 0.0001), and pTau (*p* < 0.008) showed a significant increase compared to control. Further, in SMD, pTau (*p* < 0.007), Tau, a*β*42, and APP flies (*p* < 0.0001) showed a significant increase in *Upd3* expression compared to CC flies. The effect size comparison between CC and SMD flies suggests that *Upd3* mRNA expression showed a significant increase in the abdomen of GFP (*d* = −1.13), Tau (*d* = −8.47), Tau^*R406W*^ (*d* = −1.92), pTau (*d* = −2.52), a*β*42 (*d* = −5.91), and APP (*d* = −8.14) compared to the CC condition. In the CC condition, *Dome* expression (Figure 3 G) decreased in Tau^*R406W*^ (*p* < 0.0016). In the SMD condition, pTau, a*β*42, and APP (*p* < 0.0001) showed increased expression compared to the SMD control. Further, an increase in *Dome* expression was observed in SMD Tau^*R406W*^ (*p* < 0.0009), pTau (*p* < 0.0002), a*β*42, and APP flies (*p* < 0.0001) compared to CC flies. The effect size comparison suggests *Dome* mRNA expression (Figure 3G) showed significant increase in the abdomen of Glaz/+ (*d* = −0.72), GFP (*d* = −0.26), Tau (*d* = −4.34), Tau^*R406W*^ (*d* = −7.98), pTau (*d* = −3.52), a*β*42 (*d* = −8.12), and APP (*d* = −6.63) compared to CC flies. In CC flies, *Hop* (Figure 3H) expression increased only in pTau (*p* < 0.04), whereas in SMD flies, pTau, a*β*42, and APP (*p* < 0.0001) showed a significant increase compared to SMD and CC controls. The effect size comparison suggests *Hop* mRNA expression (Figure 3 H) showed a significant increase in the abdomen of Glaz/+ in SMD flies (*d* = −0.88), GFP (*d* = −2.47), Tau (*d* = −2.70), Tau^*R406W*^ (*d* = −2.01), pTau (*d* = −4.75), a*β*42 (*d* = −11.60), and APP (*d* = −9.03) compared to CC flies. *Stat92e* (Figure 3I) in SMD flies, Tau^*R406W*^ (*p* < 0.02), and APP (*p* < 0.0001) showed a significant increase compared to control. Compared to CC flies, in SMD flies Tau (*p* < 0.001), Tau^*R406W*^, and APP (*p* < 0.0001) showed a significant increase in *Stat92e* expression. The effect size comparison between CC and SMD flies suggests *Stat92e* mRNA expression (Figure 3I) showed significant increase in the abdomen of Glaz/+ (control) in SMD flies (*d* = −0.40), GFP (*d* = −0.15), Tau (*d* = −5.20), Tau^*R406W*^ (*d* = −4.99), pTau (*d* = −1.78), a*β*42 (*d* = −1.19), and APP (*d* = −10.85) compared to CC flies. Further, we have observed a significant increase in the cell death marker *Hid* mRNA expression in the SMD condition in pTau (*p* < 0.0001), a*β*42 (*p* < 0.003), and APP (*p* < 0.0003) compared to the SMD control. Compared to CC flies, Tau (*p* < 0.0008), pTau (*p* < 0.0002), a*β*42 (*p* < 0.0001), and APP (*p* < 0.0001) showed a significant increase in SMD flies. The effect size reporting between CC and SMD flies suggests that *Hid* mRNA expression (Figure 3J) showed a significant increase in the abdomen of Glaz/+ in SMD flies (*d* = −0.21), GFP (*d* = −0.17), Tau (*d* = −4.33), Tau^*R406W*^ (*d* = −1.89), pTau (*d* = −3.73), a*β*42 (*d* = −3.65), and APP (*d* = −9.67) compared to CC flies.

We also checked the *Upd1* (SI. Figure 3A)*, Upd2* (SI. Figure 3B), *Eiger* (SI. Figure 3C)*, Imd* (SI. Figure 3D) involved in antimicrobial peptide (AMP) production. We further checked another cell death marker, *Reaper* (SI. Figure 3E). In all these genes, Tau^*R406W*^ and APP showed significant upregulation in head regions. In the body, *Upd1* expression decreased (SI. Figure 3 G) in Tau, pTau, a*β*42, and APP in CC flies, whereas in the SMD condition, these flies showed significant upregulation compared to control and CC flies. *Upd2* (SI. Figure 3 H): Tau showed reduction in CC flies compared to control, and in SMD flies, Tau, a*β*42, and APP showed significant upregulation compared to control and CC flies. In CC flies, *Eiger* (SI. Figure 3I), *Imd* (SI. Figure 3J), and *Reaper* (SI. Figure 3 K) gene expression did not show any significant change, whereas in SMD flies, except Tau^*R406W*^ for *Eiger* and a*β*42 for *Imd,* all the AD genes showed a significant upregulation in the SMD condition compared to control and CC flies. The SI. Figure 3F shows the molecular communication between cytokines and *Stat92e* for inflammatory gene expression. Effect size comparison (source data file) and *p*-values were described in the figure legends.

#### 3.1.4. Microbiome depletion aggregates sleep and circadian activity in glial AD models

Since AD pathology is associated with dysbiosis, this study aims to find the impact of dysbiosis and microbiome depletion on sleep and circadian behavioral phenotypes associated with glial-mediated AD pathology. We assessed sleep and circadian activity in control and AD transgenic flies maintained under CC and SMD conditions using the DAM. Comparisons were performed between control and AD transgenic flies within each microbiome condition and between matched CC and SMD genotypes to determine whether dysbiosis or microbiome depletion can contribute to sleep and circadian dysfunction. In our study, AD transgenic flies showed increased day activity (Figure 4B) in GFP, pTau, a*β*42, and APP (*p* < 0.0001) during the CC condition compared to control. In SMD flies, *Glaz/+,* Tau^*R406W*^ (*p* < 0.0001), and a*β*42 (*p* < 0.0071) flies showed increased day activity, and GFP, pTau, and APP (*p* < 0.0001) flies showed reduced day activity compared to CC flies. Whereas, except Tau and Tau^*R406W,*^ all the SMD flies showed reduced (*p* < 0.0001) day activity compared to SMD control flies. Effect size comparison suggests day activity in Glaz/+  (Figure 4B) (*d* = −1.41), Tau (*d* = −0.52), Tau^*R406W*^ (*d* = −1.15), a*β*42 (*d* = −1.73), and APP (*d* = −1.80) was increased and in GFP (*d* = 2.44), pTau (*d* = 2.77) reduced day activity compared CC flies. Night activity (Figure 4C) increased in GFP (*p* < 0.0001), Tau (*p* < 0.007), Tau^*R406W*^ (*p* < 0.031), and APP (*p* < 0.0008) flies (Figure 4C) compared to CC control. In SMD flies, night activity decreased in Tau (*p* < 0.0014), pTau (*p* < 0.006), a*β*42 (*p* < 0.0001), and APP (*p* < 0.0085), compared to control flies. Further we quantified Effect size comparison suggests night activity in Glaz/+ (Figure 4C) (*d* = −1.42), pTau (*d* = −0.68), Tau (*d* = −0.53), increased and in GFP (*d* = 0.43), Tau^*R406W*^ (*d* = 0102), a*β*42 (*d* = 0.93), and APP (*d* = 0.30) decreased compared CC flies. Total activity (Figure 4D) increased in GFP and APP (*p* < 0.0001) and was reduced in a*β*42 (*p* < 0.015) flies compared to control in CC flies. In SMD flies, Tau (*p* < 0.0009), pTau, a*β*42, and APP (*p* < 0.0001) flies showed reduced total activity compared to control. In SMD flies, control and Tau^*R406W*^ (*p* < 0.0001) flies showed increased total activity, and GFP, APP (*p* < 0.0001) flies showed reduced total activity (Figure 4D) compared to CC flies. Effect size comparison suggests total activity in Glaz/+ (Figure 4D) (*d* = −1.50), Tau (*d* = −0.28), Tau^*R406W*^ (*d* = −0.94), a*β*42 (*d* = −0.44) increased, and in GFP (*d* = 1.87), pTau (*d* = 0.81), and APP (*d* = 1.80) flies decreased compared CC flies.

**Figure 4.**
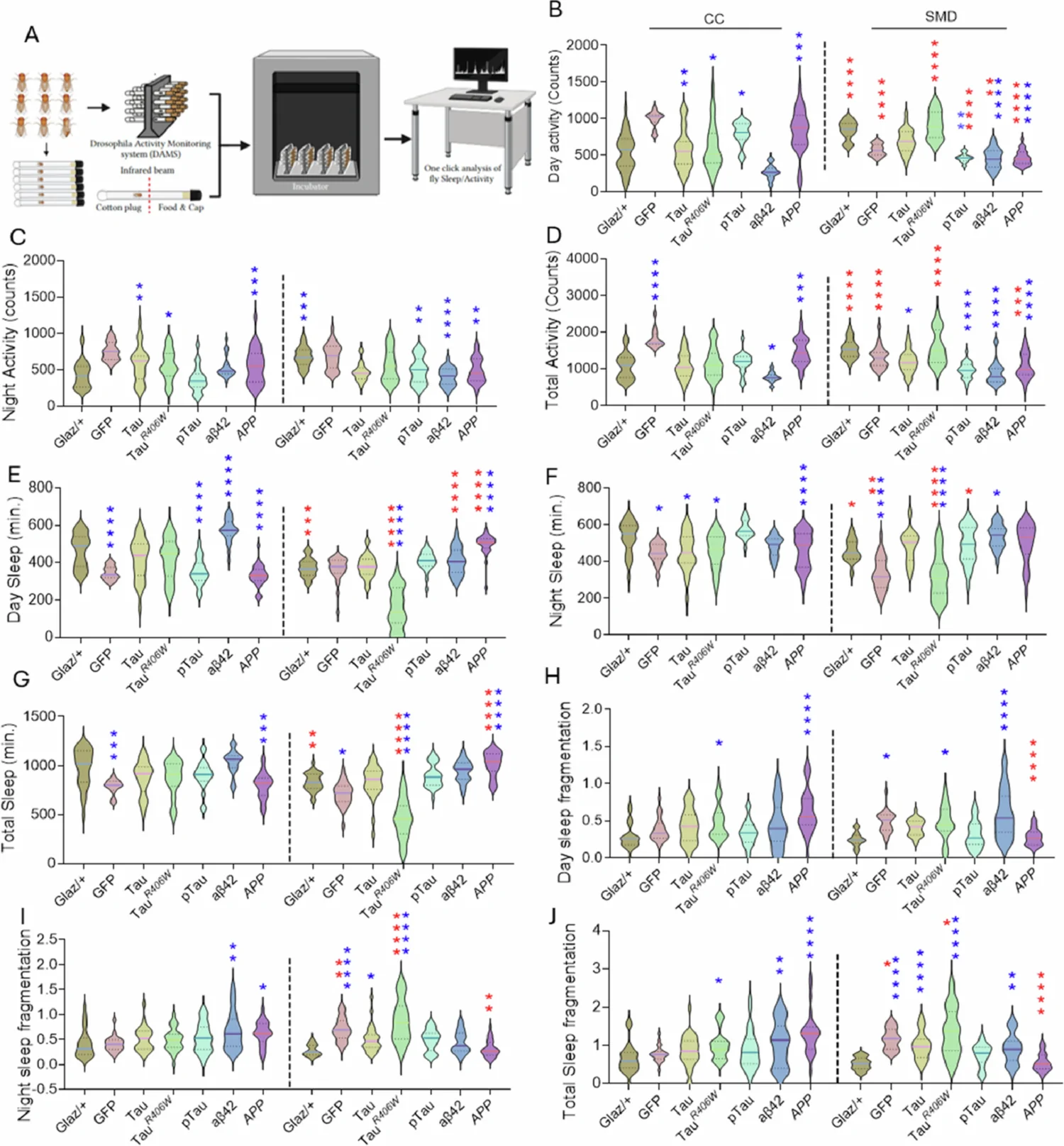
Microbiome depletion aggregates sleep-circadian activity in humanized AD *Drosophila* models: The sleep/activity rhythms of CC and SMD flies were measured across 24 h for 3 consecutive days at 3 weeks of age. (A). Schematic representation of the sleep/activity assay using *Drosophila* activity monitoring systems and one-click analysis. (B). Day activity, (C). Night activity, (D). Total activity. (E). Day sleep, (F). Night sleep, (G). Total sleep. (H). Day sleep fragmentation, (I). Night sleep fragmentation (J). Total sleep fragmentation. Blue asterisks represent the comparison between Glaz/+  and AD genotypes in each condition, and red asterisks represent the comparison between identical genotypes reared under CC and SMD conditions. Two-way ANOVA to establish significance, followed by Sidak’s multiple comparisons test to assess the effects of genotype and microbiome condition. *n* = 3 biological replicates in every genotype and condition (each replicate consists of 16 flies) and *p*-values: * < 0.05, ** < 0.01, *** < 0.001, **** < 0.0001. Raw data and specific *p-*values are provided in the source data file.

Day sleep (Figure 4E) was reduced in GFP, pTau, and APP (*p* < 0.0001) and increased in a*β*42 (*p* < 0.0001) flies compared to control in CC flies. In SMD flies’ control (*p* < 0.0008), Tau^*R406W*^ and a*β*42 (*p* < 0.0001) showed reduced day sleep, and APP (*p* < 0.0001) showed increased day sleep compared to CC flies. In SMD flies, APP (*p* < 0.0001) showed increased day sleep, and Tau^*R406W*^ (*p* < 0.0001) showed reduced day sleep compared to SMD control flies, with no significant difference in Tau and pTau flies. Further, we quantified the effect size comparison between CC and SMD flies, suggesting SMD flies showed significant changes in day sleep of Glaz/+ (Figure 4E) (*d* = 1.26), GFP (*d* = 0.38), Tau (*d* = 0.43), Tau^*R406W*^ (*d* = 2.43), pTau (*d* = −0.92), a*β*42 (*d* = 2.43), and APP (*d* = −2.90) compared with CC flies. Night sleep (Figure 4F) was significantly reduced in GFP (*p* < 0.028), Tau (*p* < 0.015), Tau^*R406W*^ (*p* < 0.025), and APP (*p* < 0.046) flies, except in pTau and a*β*42 flies compared to control flies. In SMD flies, control (*p* < 0.03), GFP, Tau^*R406W*^ (*p* < 0.0001), and pTau (*p* < 0.016) showed reduced night sleep compared to CC flies. Among the SMD flies, night sleep was reduced in GFP (*p* < 0.0008) and Tau^*R406W*^ (*p* < 0.0001) flies and increased in a*β*42 (*p* < 0.021) flies compared to control. Effect size comparison suggests SMD flies showed significant change in night sleep in Glaz/+  (Figure 4F) (*d* = 0.98), GFP (*d* = 1.46), Tau (*d* = −0.32), Tau^*R406W*^ (*d* = 1.31), pTau (*d* = 1.03), a*β*42 (*d* = −0.90), and APP (*d* = −0.36) compared to CC flies. Total sleep (Figure 4 G) was reduced in GFP (*p* < 0.0004) and APP (*p* < 0.0001) compared to control in CC flies. In SMD flies’ control (*p* < 0.0041), Tau^*R406W*^ (*p* < 0.0001) showed reduced total sleep, and APP (*p* < 0.0001) showed increased total sleep compared to CC flies. Among the SMD flies, Tau^*R406W*^ (*p* < 0.0001) showed reduced total sleep, and APP (*p* < 0.0001) showed increased total sleep compared to SMD control flies. Effect size comparison showed significant change in *Glaz/+*  (*d* = 0.95), GFP (*d* = 0.68), Tau (*d* = 0.22), Tau^*R406W*^ (*d* = 2.16), pTau (*d* = 0.02), a*β*42 (*d* = 0.77), and APP (*d* = −1.66) compared to CC flies.

Day bout numbers (SI. Figure 4A) increased in APP compared to control in CC flies. In SMD flies, GFP, Tau, and a*β*42 showed increased day bout numbers compared to SMD control, and Tau^*R406W*^ and APP showed reduced day bout numbers compared to CC flies. Night bout numbers (SI. Figure 4B) increased in pTau compared to Control in CC flies. In SMD flies, GFP and Tau^*R406W*^ showed increased night bout numbers compared to SMD control, and APP showed reduced night bout numbers compared to its CC counterpart. Total bout numbers (SI. Figure 4C) increased in a*β*42 and APP flies compared to control in CC flies, and APP in SMD flies showed a reduction compared to APP in CC flies. Among the SMD flies, GFP, Tau, and pTau showed increased total bout numbers compared to SMD control flies. Day bout length (SI. Figure 4D) was reduced in all AD flies in CC compared to control, except a*β*42; in SMD flies, Tau^*R406W*^ showed reduced and APP flies showed increased day bout length compared to CC flies. Among the SMD flies, GFP, Tau, Tau^*R406W*^, and a*β*42 showed significant reduction in day bout length compared to SMD control flies. Night bout length (SI. Figure 4E) was reduced in all AD flies compared to control in CC flies, and in SMD flies, GFP showed a reduction, and a*β*42 and APP showed increased night bout length compared to CC flies. Among the SMD flies, GFP, Tau, Tau^*R406W*^, and pTau flies showed reduced night bout length compared to SMD control flies. Total bout length (SI. Figure 4F) was reduced in all AD flies except a*β*42 in CC flies; in SMD flies, Tau^*R406W*^ showed reduced, and APP showed increased total bout length compared to CC flies. Within SMD flies, GFP, Tau, and Tau^*R406W*^ showed significant reduction in total bout length compared to SMD control flies. Effect size comparisons (Cohen’s d) for all matched CC and SMD fly genotypes were calculated and represented in the source data for Figure 4 & S.I. Figure 4.

Day sleep fragmentation (Figure 4H) increased in Tau^*R406W*^ (*p* < 0.02) and APP (*p* < 0.0001) flies in the CC condition compared to control, and in SMD flies, APP (*p* < 0.0001) showed a reduction compared to the CC condition. In SMD flies, Tau^*R406W*^ (*p* < 0.003), GFP, and a*β*42 (*p* < 0.0001) flies showed increased day sleep fragmentation compared to SMD control flies. Effect size comparison showed significant changes in day sleep fragmentation of Glaz/+  (Figure 4H) (*d* = 0.29), GFP (*d* = −0.68), Tau (*d* = 0.07), Tau^*R406W*^ (*d* = 0.05), pTau (*d* = 0.07), a*β*42 (*d* = −0.46), and APP (*d* = 1.50) compared with CC flies. Night sleep fragmentation (Figure 4I) increased in a*β*42 (*p* < 0.035) and APP (*p* < 0.049) in CC flies and in SMD flies APP (*p* < 0.021) showed reduction compared to controls and Tau (*p* < 0.024), GFP (*p* < 0.0001), Tau^*R406W*^ (*p* < 0.0001) in SMD condition showed increased night sleep fragmentation compared to CC flies. Among the SMD flies, GFP (*p* < 0.001) and Tau^*R406W*^ (*p* < 0.0001) flies showed increased night-sleep fragmentation compared to SMD control. The effect size comparison between CC and SMD flies showed significant changes in night sleep fragmentation in Glaz/+ (Figure 4I) (*d* = 0.61), GFP (*d* = −1.41), Tau (*d* = 0.04), Tau^*R406W*^ (*d* = −1.11), pTau (*d* = 0.19), a*β*42 (*d* = 0.74), and APP (*d* = 1.49) compared with CC flies. Total sleep fragmentation (Figure 4J) showed significant increase in Tau^*R406W*^ (*p* < 0.021), a*β*42 (*p* < 0.0027) and APP (*p* < 0.0001) in CC flies compared to control, whereas in SMD flies increase in GFP (*p* < 0.01), Tau^*R406W*^ (*p* < 0.02), and decrease in APP (*p* < 0.0001) flies compared to CC flies. Among the SMD flies, Tau (*p* < 0.0003), GFP, Tau^*R406W*^ (*p* < 0.0001), and a*β*42 (*p* < 0.0045) showed increased total sleep fragmentation compared to SMD control flies. Further, the effect size comparison between CC and SMD flies suggests SMD flies showed significant changes in total sleep fragmentation in Glaz/+  (Figure 4J) (*d* = 0.46), GFP (*d* = −1.58), Tau (*d* = 0.37), Tau^*R406W*^ (*d* = −0.67), pTau (*d* = 0.35), a*β*42 (*d* = 0.28), and APP (*d* = 1.72) compared CC flies.

#### 3.1.5. Microbiome depletion exacerbates learning and memory impairment in glial AD models

To determine whether microbiome depletion influences learning and memory phenotypes during glial-mediated AD pathology, we assessed olfactory learning behavior under the influence of an electric shock training paradigm in control and AD transgenic flies maintained under CC and SMD conditions. Figure 5A is the schematic representation of the memory assay setup. In CC flies, Figure 5B represents the average number of flies preferring the shock odor before and after training/electric shock. Reduction in odor preference after electric shock suggests better memory. In our study *Glaz*/+  (53.57/22.73), GFP (59.46/34.38), Tau (60/33) flies showed reduced preference to same odor after electric shock whereas Tau^*R406W*^ (50/53.85), pTau (56.41/54.29), a*β*42 (63.64/60.53) and APP (54.35/54.29) flies showed no change in odor preference/did not learn even after electric shock. This data suggests memory impairment in Tau^*R406W*^, pTau, a*β*42, and APP in CC flies. In SMD flies, we found a significant memory impairment in all the AD-linked genotypes (Figure 5C) compared to control (60/31.25) and GFP (64/28) flies in Tau (63.64/62.5), Tau^*R406W*^ (65/56.25), pTau (60/58.33), a*β*42 (66.67/61), and APP (50/52.38). We then calculated the olfactory performance index based on the odor choice (Figure 5D), where negative values represent better performance/no memory impairment and positive values represent severe memory impairments. We represent the CC/SMD olfactory preference index for *Glaz/+* (−0.47/−0.33), GFP (−0.26/−0.28), Tau (−0.16/0.11), Tau^*R406W*^ (0.11/0.21), pTau (0.13/0.21), a*β*42 (0.14/0.45), and APP (0.28/0.11) AD genotypes. Further, we quantified the effect size comparison between CC and SMD flies, which suggests that SMD flies further exhibit memory deficits in Glaz/+ (*d* = −1.36), Tau (*d* = −1.75), pTau (*d* = −0.52), aβ42 (*d* = −0.59), and no improvement in GFP (*d* = 0.54), Tau^*R406W*^ (*d* = 0.99), and APP (*d* = 0.96) compared with CC flies.

**Figure 5.**
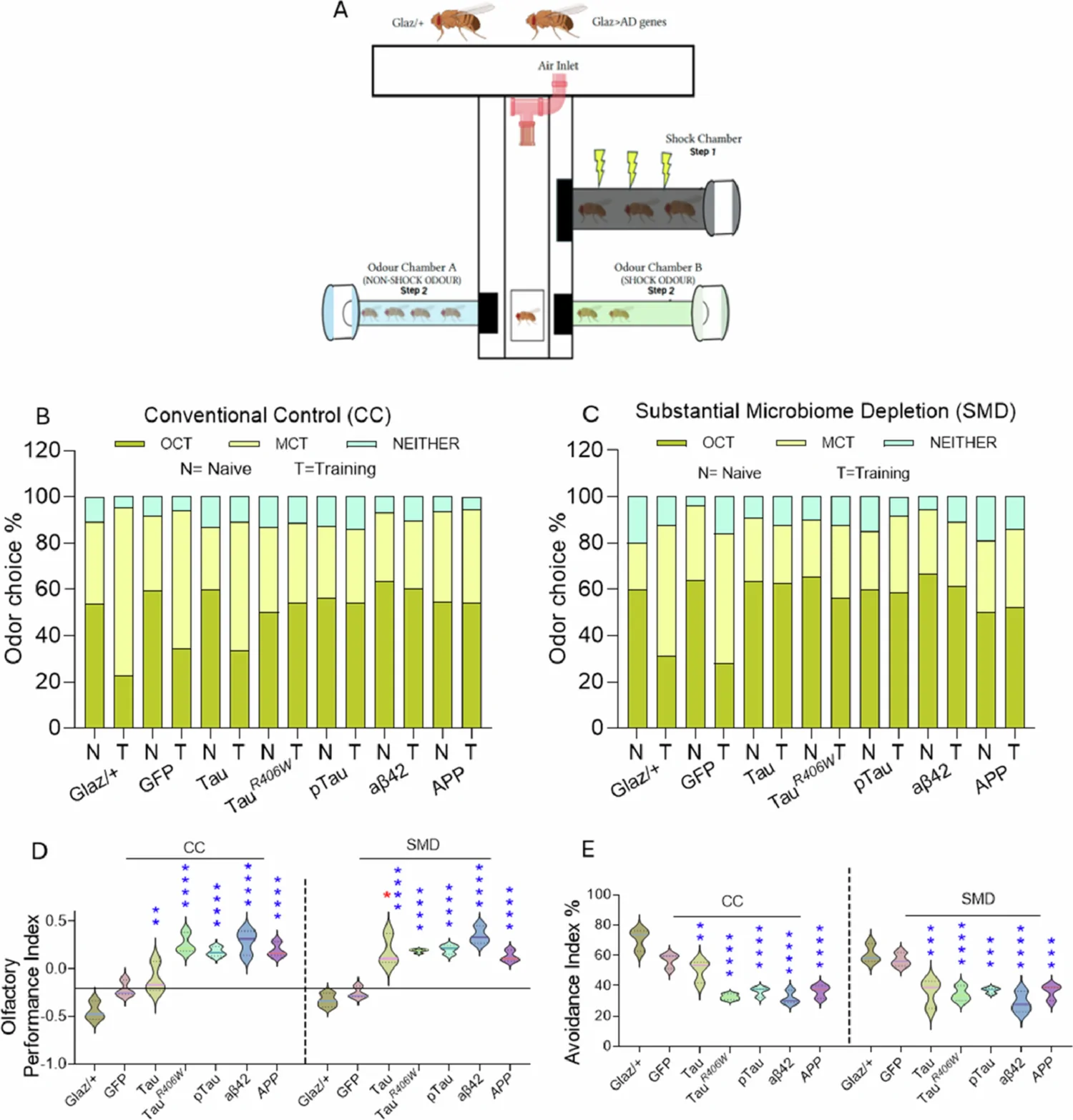
Depletion of microbiomes deteriorates the memory impairment linked with AD. (A). Schematic representation of the memory assay for *Drosophila* (B). Comparison of odor choice% between Naïve (*N*)-before shock and Training (T) after shock in conventional control (CC)/with microbiome AD and control flies. If flies still prefer the shock odor, it implies memory impairment. (C). Odor choice % in antibiotic-treated (SMD) AD and control flies (D). Olfactory performance index % showed a significant increase in Tau (*p* < 0.01), Tau^*R406W*^, pTau, a*β*42, and APP (*p* < 0.0001) in CC flies; in SMD, all the AD flies showed a significant increase (*p* < 0.0001) compared to control, and Tau(*p* < 0.05) showed a significant increase compared to CC flies. (E). Avoidance index % was significantly reduced in CC (*p* < 0.0001) and SMD (*p* < 0.0001) AD flies compared to controls. Blue asterisks represent the comparison between Glaz/+  and AD genotypes in each condition, and red asterisks represent the comparison between identical genotypes reared under CC and SMD conditions. In the memory assay for panels B and C, we used a chi-square test; in panels D and E, a two-way ANOVA with Sidak’s multiple comparison test was used. *n* = 3 biological replicates in every genotype and condition (each replicate consists of 30–40 flies), and *p*-values: * < 0.05, ** < 0.01, *** < 0.001, **** < 0.0001. Raw data and specific *p-*values are provided in the source data file.

To further evaluate behavioral performance, we calculated the avoidance index% (Figure 5E), where higher values represent better memory performance and lower values represent memory impairment. The avoidance index% for AD flies: *Glaz/+* (73.52/58.33), GFP (59.6/61.9), Tau (53.3/38.8), Tau^*R406W*^ (32.4/30), pTau (37.8/34.7), a*β*42 (37.12/22.72), and APP (31.57/38.88). Further we quantified the effect size comparison between CC and SMD flies suggests SMD flies showed no improvement or further decline in avoidance index% in Glaz/+ (*d* = 1.50), GFP (*d* = −0.05), Tau (*d* = 1.72), Tau^*R406W*^ (*d* = −0.49), pTau (*d* = −0.33), aβ42 (*d* = 0.52), and APP (*d* = 0.07) compared CC flies. The olfactory preference assay showed clear learning/memory phenotypes in some genotypes in CC flies, and the SMD flies showed exacerbated memory loss in all AD models. The performance index and avoidance index% further confirm the possible impact of loss of the gut microbiome, further worsening the cognitive decline in AD models.

To understand the influence of microbiome depletion on locomotor behavior, we further assessed negative geotaxis in male (SI. Figure 5A–F) and female (SI. Figure 6A–F) flies under CC and SMD conditions. The line graph represents the climbing distance of each genotype from 0 to 9 seconds. We have observed a significant reduction in locomotory performance in SMD, GFP, Tau^*R406W*^, and pTau flies compared to CC GFP, Tau^*R406W*^, and pTau flies. No significant difference in Tau SMD flies compared to CC counterparts. But a*β*42 and APP showed a significant increase in SMD flies compared to CC flies in both males and females. In females, Tau and Tau^*R406W*^ showed significant changes in locomotor performance in SMD flies compared to CC flies.

**Figure 6.**
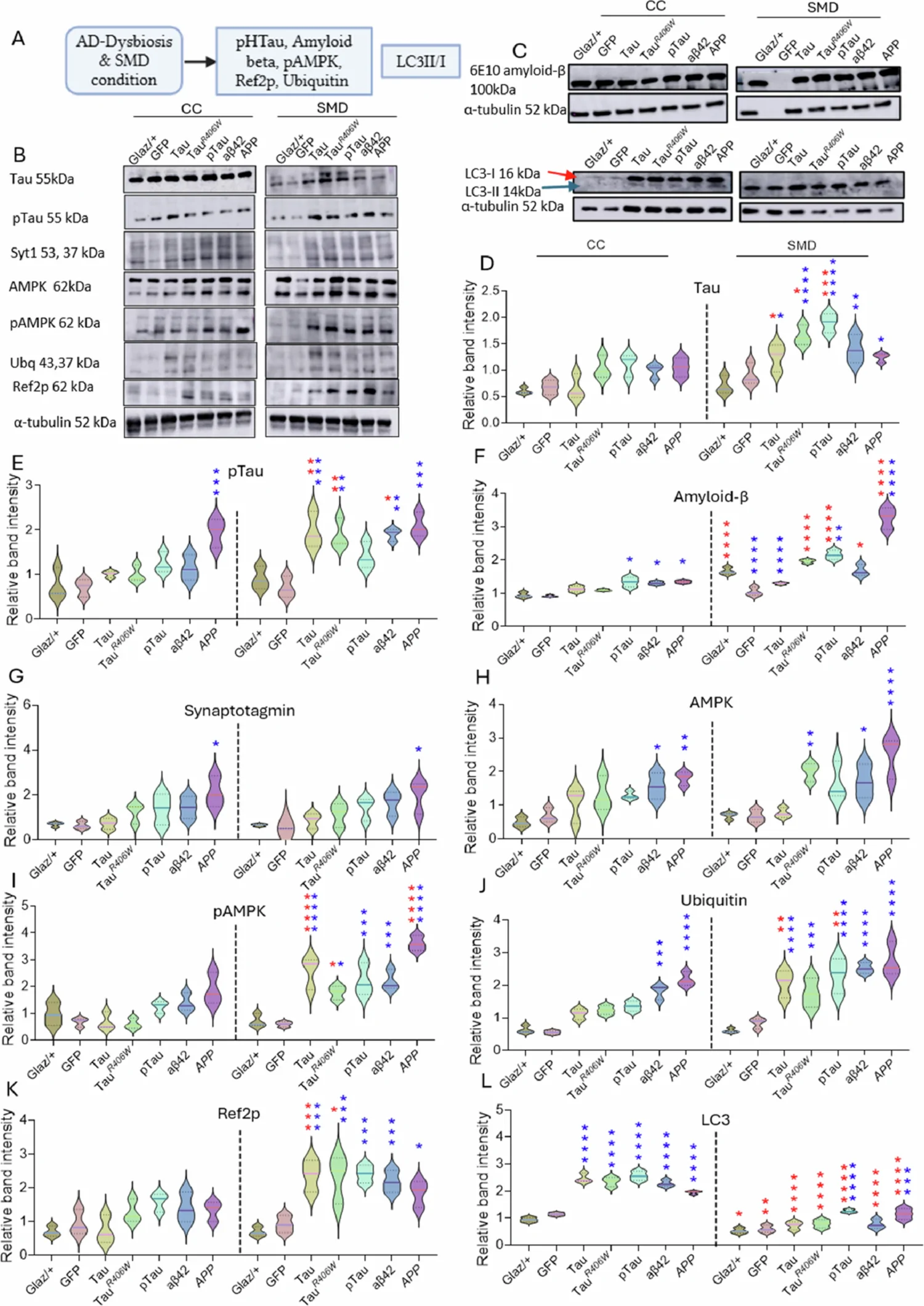
Depletion of microbiomes resulted in upregulation of amyloid-*β* and phospho-Tau through AMPK phosphorylation and impaired autophagy-mediated signaling. Western blot analysis to quantify the abundance of targeted proteins in the head. B and C are representative blots. (A). Representing the impact of dysbiosis and SMD conditions on AD pathology. (D). Tau, (E). Phospho-Tau (F). a*β*42, (G). Syt1 (synaptotagmin), (H). AMPK, (I). Phospho-AMPK (J). Ubiquitinated proteins (K). Ref(2)p (p62 homolog of *Drosophila*), (L). LC3. All the representative blots (B and C) were shown along with the internal control *α*-tubulin. Blue asterisks represent the comparison between Glaz/+  and AD genotypes in each condition, and red asterisks represent the comparison between identical genotypes reared under CC and SMD conditions. Statistical analysis was performed with Two-way ANOVA to establish significance, followed by Sidak’s multiple comparisons test to assess the effects of genotype and microbiome condition. *n* = 3 biological replicates in every genotype and condition (each replicate consists of 10–15 flies), and *p*-values: * < 0.05, ** < 0.01, *** < 0.001, **** < 0.0001. Raw data and specific *p-*values are provided in the source data file.

#### 3.1.6. Microbiome depletion enhanced the aggregation of AD-associated protein by impairing autophagy and proteostasis-related pathways in glial AD models

Further, we investigate whether microbiome depletion influences molecular pathways associated with metabolic homeostasis and protein quality control during glial-mediated AD pathology. We quantified proteins involved in cellular energy sensing (AMPK and phospho-AMPK), proteostasis (Ref(2)P, ubiquitinated proteins, and GABARAP or LC3I/II), synaptic protein abundance (Synaptotagmin), and AD pathology (Tau, phospho-Tau, and amyloid-*β*) in control and AD transgenic flies maintained under CC and SMD conditions. This comparison helps us to understand molecular changes associated with AD transgene expression independent of microbiome depletion, allowing evaluation of both factors across all genotypes.

Total AMPK levels were quantified in SMD and CC flies to determine whether microbiome depletion modifies cellular energy-sensing pathways during AD pathology (Figure 6H). In CC, a*β*42 (*p* < 0.02) and APP (*p* < 0.003) flies; in SMD, Tau^*R406W*^ (*p* < 0.003), a*β*42 (*p* < 0.04), and APP (*p* < 0.0001) flies showed a significant increase compared to controls. Effect size comparison between CC and SMD flies suggests a significant increase in AMPK band intensity in Glaz/+ (*d* = −1.48), GFP (*d* = −0.05), Tau (*d* = 0.74), Tau^*R406W*^ (*d* = −1.65), pTau (*d* = −0.91), a*β*42 (*d* = −0.31), and APP (*d* = −1.47) compared with CC flies. pAMPK (Figure 6I) in SMD condition Tau (*p* < 0.003), Tau^*R406W*^ (*p* < 0.032), pTau (*p* < 0.0007), a*β*42 (*p* < 0.0007), and APP (*p* < 0.0001) flies showed a significant increase compared to control flies. In addition, we observed a significant increase in pAMPK in Tau (*p* < 0.0001), Tau^*R406W*^ (*p* < 0.012), and APP (*p* < 0.0001) compared to CC flies. Effect size analysis showed a significant microbiome-dependent effect across multiple AD genotypes, including Glaz/+ (*d* = 0.73), GFP (*d* = 0.59), Tau (*d* = −3.90), Tau^*R406W*^ (*d* = −5.07), pTau (*d* = −2.14), a*β*42 (*d* = −2.27), and APP (*d* = −3.72) compared to CC flies. This suggests enhanced AMPK phosphorylation^46^ ^,^ ^47^ and an altered cellular energy-sensing response during AD pathology in SMD condition in flies.

Since AMPK signaling has been implicated in the regulation of protein quality-control pathways, we next quantified ubiquitinated proteins and Ref(2)P to determine whether microbiome depletion was associated with alterations in proteostasis during AD pathology.^48^ ^,^ ^49^ The ubiquitinated protein (Figure 6J) band intensity in CC flies a*β*42 (*p* < 0.0007), and APP (*p* < 0.0001) showed a significant increase compared to control. In SMD flies, Tau (*p* < 0.0093) and pTau (*p* < 0.0083) showed A significant increase compared to CC flies. In SMD Tau^*R406W*^ (*p* < 0.0009), Tau, pTau, a*β*42, and APP (*p* < 0.0001) flies showed a significant increase compared to SMD control flies. The effect size comparison between CC and SMD flies suggests SMD flies showed a significant increase in ubiquitinated protein band intensity in Glaz/+ (*d* = 0.28), GFP (*d* = −2.36), Tau (*d* = −2.95), Tau^*R406W*^ (*d* = −1.58), pTau (*d* = −2.40), a*β*42 (*d* = −3.62), and APP (*d* = −1.40) compared with CC flies. We then examined proteins associated with intracellular protein quality control Ref(2)p/p62, which directs damaged proteins to the autophagosome.^50^ ^,^ ^51^ In our study, we did not observe any significant increase in CC flies compared to control (Figure 6K). In contrast, Tau (*p* < 0.0002) and Tau^*R406W*^ (*p* < 0.041) flies showed an increased accumulation of Ref(2)p in SMD compared to CC flies. Whereas Tau (*p* < 0.0003), Tau^*R406W*^ (*p* < 0.0002), pTau (*p* < 0.0001), a*β*42 (*p* < 0.0007), and APP (*p* < 0.014) flies showed a significant increase in Ref2p compared to SMD control flies. The effect size comparison between CC and SMD flies suggests a significant microbiome-dependent impact on Glaz/+  (*d* = 0.057), GFP (*d* = 0.076), Tau (*d* = −3.66), Tau^*R406W*^ (*d* = −1.86), pTau (*d* = −3.02), a*β*42 (*d* = −1.98), and APP (*d* = −1.47) compared with CC flies. To confirm whether accumulation of ubiquitinated proteins and Ref(2)p was influenced by autophagy-associated proteins, we quantified LC3 protein expression in CC and SMD flies (Figure 6L), we find a significant increase in Tau, Tau^*R406W*^, pTau, a*β*42 and in APP (*p* < 0.0001) significantly in CC flies Compared to control. In SMD flies pTau (*p* < 0.0001) and APP (*p* < 0.0007) showed a significant increase compared to control. Between CC and SMD flies, we found a significant reduction in Glaz/+ (*p* < 0.03), GFP (*p* < 0.002), Tau, Tau^*R406W*^, pTau, a*β*42, and in APP (*p* < 0.0001) compared to CC flies. The effect size comparison between CC and SMD flies suggests LC3 was reduced significantly in SMD, Glaz/+ (*d* = 4.21), GFP (*d* = 5.26), Tau (*d* = 10.5), Tau^*R406W*^ (*d* = 9.36), pTau (*d* = 9.77), a*β*42 (*d* = 9.86), and APP (*d* = 5.22) compared CC flies. LC3-II/I (Atg8b/Atg8a/) ratio in CC and SMD flies (S.I. Figure 7 C). We found a negligible LC3-II protein band in SMD. At the same time, CC flies LC3-II band is apparently visible in all the samples. Then we calculated the LC3-II/LC3-I ratio between genotypes and groups. Compared to Glaz/+, Tau, Tau^*R406W*^, pTau, and a*β*42 showed a significant decrease (*p* < 0.0001) in CC, and Tau^*R406W*^ showed a significant decrease (*p* < 0.03) in SMD in each group. The effect size comparison between CC and SMD flies suggests in LC3-II/I ratio was reduced significantly in Glaz/+ (*d* = 3.52), GFP (*d* = 4.44), Tau (*d* = −0.55), Tau^*R406W*^ (*d* = 0.31), pTau (*d* = 0.96), a*β*42 (*d* = −1.22), and APP (*d* = 4.82) compared CC flies.

**Figure 7.**
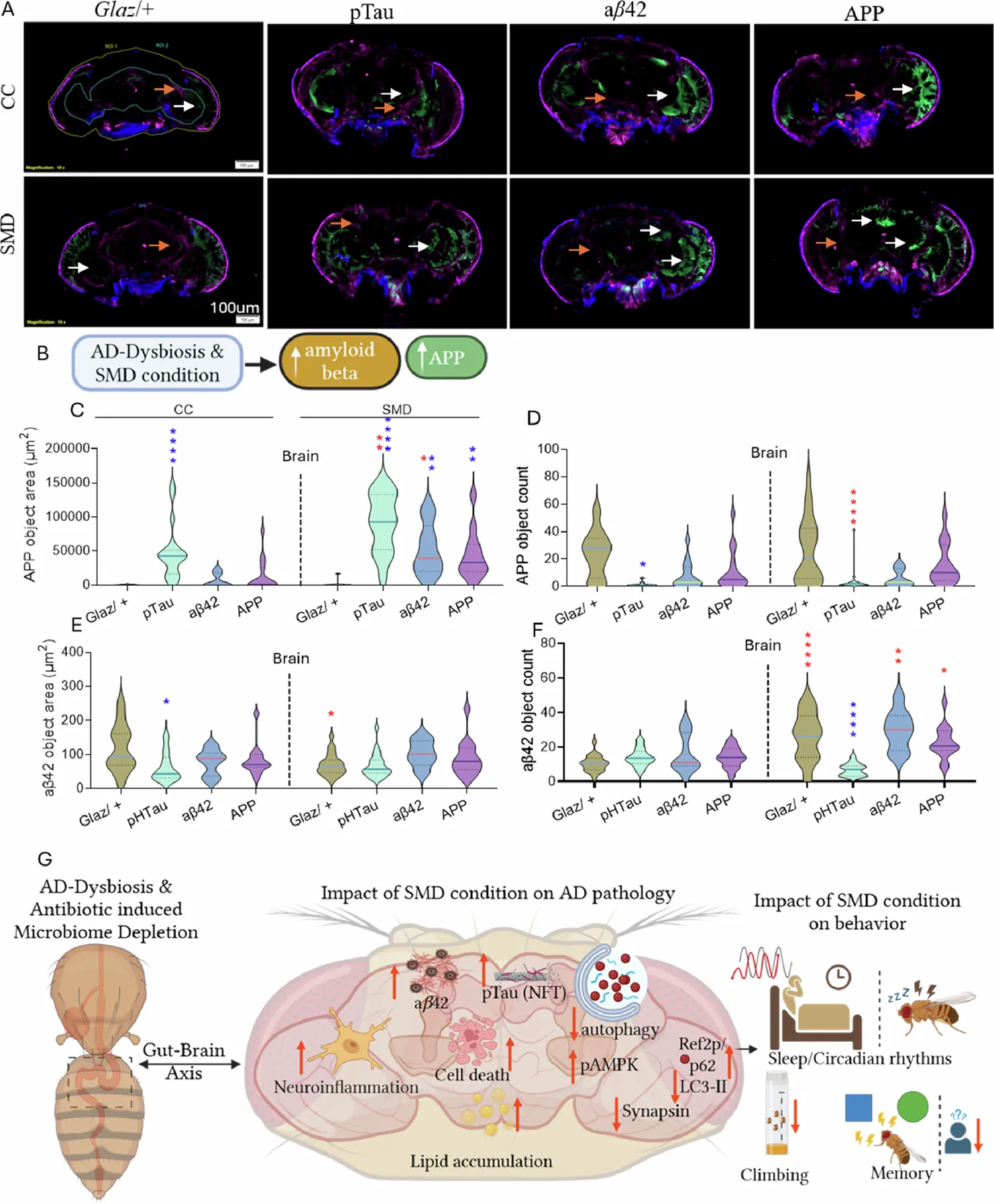
Effects of microbiome depletion and sleep/microbiome disruption on Alzheimer’s disease (AD)-related pathology, autophagy, metabolism, and behavioral outcomes in humanized AD *Drosophila* models. (A–B) Representative brain images showing amyloid-*β*42 (a*β*42; orange) and amyloid precursor protein (APP; green) staining under the indicated genotypes and experimental conditions. The arrows indicate representative staining signals. (A) Representative brain regions and regions of interest (ROIs). (B) Representative staining patterns for a*β*42 and APP. Scale bar, 100 μm. (C–F) Quantification of APP object area (C), APP object count (D), a*β*42 object area (E), and a*β*42 object count (F) in the brain. Blue asterisks indicate comparisons between Glaz/+  and AD genotypes within the same condition, whereas red asterisks indicate comparisons between the control condition (CC) and the sleep/microbiome disruption (SMD) condition within the same genotype. (G) Schematic illustration of the proposed effects of AD-associated dysbiosis and SMD on AD-related pathology and physiological function. The illustration summarizes neuroinflammation, lipid accumulation, a*β*42 and phosphorylated tau (pTau/NFT) accumulation, altered autophagy, and cell death. It also highlights changes in synapsin (synaptic function), phosphorylated AMPK (pAMPK; metabolic signaling), and Ref(2)P/p62 and LC3-II (autophagy-associated markers), alongside potential effects on behavioral outcomes, including sleep/circadian rhythms, climbing, and memory. Statistical significance was assessed using two-way ANOVA followed by Sidak’s multiple-comparisons test to evaluate genotype and microbiome-condition effects. *n* = 5 per genotype and condition; three brain tissue sections were analyzed per fly. Significance levels: *p* < 0.05, *p* < 0.01, *p* < 0.001, and *p* < 0.0001. Raw data and exact *p*-values are provided in the Source Data file.

Based on the results obtained from proteostasis (ubiquitinated proteins, Ref2p) and autophagy-associated proteins (LC3II/I ratio), we then proceeded to check whether these changes coincided with increased accumulation of AD-associated pathological proteins. Tau protein band intensity (Figure 6D) in Tau (*p* < 0.032), Tau^*R406W*^, pTau (*p* < 0.0001), a*β*42 (*p* < 0.0026), and APP (*p* < 0.037) flies showed a significant increase in the SMD condition compared to the SMD control. In SMD flies, Tau (*p* < 0.015), Tau^*R406W*^ (*p* < 0.011), and pTau (*p* < 0.0005) showed a significant increase in Tau protein band intensity compared to CC Tau, Tau^*R406W*^, and pTau flies. The effect size comparison between CC and SMD flies suggests a significant increase in tau accumulation in Glaz/+  (*d* = −0.73), GFP (*d* = −1.28), Tau (*d* = −2.23), Tau^*R406W*^ (*d* = −2.95), pTau (*d* = −3.92), a*β*42 (*d* = −1.94), and APP (*d* = −1.30) compared to CC flies. We then checked pTau (Figure 6E); in CC flies, APP (*p* < 0.0002) showed a significant increase compared to CC control flies. In SMD flies, Tau (*p* < 0.0009), Tau^*R406W*^ (*p* < 0.0024), a*β*42 (*p* < 0.002), and APP (*p* < 0.0002) showed a significant increase in pTau band intensity compared to SMD control flies. In Tau (*p* < 0.0029), Tau^*R406W*^ (*p* < 0.0074), and a*β*42 (*p* < 0.033) flies, we found a significant increase in pTau compared to CC flies. The effect size comparison between CC and SMD flies suggests a significant increase in pTau protein band intensity in Glaz/+  (*d* = −0.48), GFP (*d* = 0.02), Tau (*d* = −3.33), Tau^*R406W*^ (*d* = −3.33), pTau (*d* = −0.59), a*β*42 (*d* = −3.36), and APP (*d* = −0.45) in SMD flies compared to CC flies. The amyloid-*β* protein band intensity (Figure 6F) is increased in pTau, a*β*42, and APP (~p < 0.02) in CC flies compared to control. In SMD flies compared to control and pTau (*p* < 0.002), APP (*p* < 0.0001) compared to SMD control flies. In the SMD condition, Tau^*R406W*^, pTau (*p* < 0.0001), and a*β*42 (*p* < 0.01) showed a significant increase compared to CC flies. The effect size comparison between CC and SMD flies suggests a significant increase in a*β*42 protein band intensity in Glaz/+ (*d* = −7.44), GFP (*d* = −1.33), Tau (*d* = −2.48), Tau^*R406W*^ (*d* = −18.78), pTau (*d* = −5.71), a*β*42 (*d* = −2.89), and APP (*d* = −8.34) in SMD flies compared with CC flies. Further amyloid-*β* protein aggregates were tested using western blot (S.I. Figure 7A–B), suggesting that pTau and APP flies showed a significant increase in amyloid-*β* protein aggregates during the mycobiome depletion state.

The accumulation of Tau, pTau, and a*β*42 is closely associated with synaptic dysfunction and disease progression in AD; we quantified synaptotagmin (Syt1), a key synaptic vesicle protein involved in neurotransmitter release^52^ (Figure 6 G). We observed a significant increase in band intensity of Syt1 in APP (*p* < 0.014) in CC and in SMD flies (*p* < 0.027) compared to control. The effect size comparison between CC and SMD flies suggests no significant variation between CC and SMD flies in Syt1 protein band intensity in Glaz/+ (*d* = 0.32), GFP (*d* = 1.19), Tau (*d* = −0.45), Tau^*R406W*^ (*d* = −0.17), pTau (*d* = −0.09), aβ42 (*d* = −0.39), and APP (*d* = −0.172) compared to CC flies. To understand the possible connection between inflammatory signaling and AD-associated pathology, we tested a*β*42, APP, Tau, phospho-Tau, and the inflammatory marker STAT5A/Stat92e in a UPD3 (IL6) overexpression fly model. UPD3 overexpression fly line was crossed with the Glaz-Gal4 driver to facilitate glial-specific expression. Our western blot analysis results suggest a significant correlation between increased inflammation and AD-associated proteins (SI. Figure 8A–F).

#### 3.1.7. Immunofluorescence analysis revealed that microbiome depletion enhanced amyloid-*β* and APP accumulation in the brain and abdomen

To further confirm that AD-associated protein aggregates also affect cytology, we performed immunofluorescence analysis for amyloid-*β* and APP proteins using pTau, a*β*42, and APP in flies in CC and SMD conditions (Figure 7 and S.I. Figure 9). APP object area (Figure 7C) showed a significant increase in pTau in both CC and SMD conditions (*p* < 0.0001) compared to respective controls. Compared to CC flies, pTau (*p* < 0.002) and a*β*42 (*p* < 0.01) showed a significant increase in the SMD condition. Whereas in the SMD condition, a*β*42 and APP (*p* < 0.003) showed a significant increase. The effect size comparison between CC and SMD flies suggests significant variation in APP object area in the brain between CC and SMD flies in Glaz/+ (*d* = −0.54), pTau (*d* = −0.54), aβ42 (*d* = −1.47), and APP (*d* = −0.98) compared with CC flies. APP object counts in the brain (Figure 7D) decreased in pTau (*p* < 0.04) in CC compared to control and increased in SMD (*p* < 0.0001) compared to pTau in CC. The effect size comparison between CC and SMD flies suggests significant variation in APP object count in the brain between CC and SMD flies in Glaz/+ (*d* = −0.097), pTau (*d* = −0.27), a*β*42 (*d* = 0.25), and APP (*d* = −0.31) compared with CC flies. a*β*42 object area (Figure 7E) decreased in pTau (*p* < 0.03) and SMD Glaz/+ (*p* < 0.002) compared to CC control. The effect size comparison between CC and SMD flies suggests significant variation in a*β*42 object area in the brain between CC and SMD flies in Glaz/+ (*d* = 1.06), pTau (*d* = −0.14), a*β*42 (*d* = −0.66), and APP (*d* = −0.14) compared with CC flies. a*β*42 object counts (Figure 7F) significantly increased in SMD control, (*p* < 0.0001), a*β*42 (*p* < 0.003) and APP (*p* < 0.01) compared to flies under CC condition. The effect size comparison between CC and SMD flies suggests significant variation in a*β*42 object count in the brain between CC and SMD flies in Glaz/+ (*d* = 1.79), pTau (*d* = 1.69), a*β*42 (*d* = −1.21), and APP (*d* = −1.13) compared to CC flies. In SMD, pTau showed a significant decrease (*p* < 0.0001) in a*β*42 object count compared to control. In the abdomen, APP object count increased in a*β*42 (*p* < 0.03) compared to control in CC and SMD conditions (S.I. Figure 9C). a*β*42 object count (S.I. Figure 9D) increased in a*β*42, APP (*p* < 0.0001) compared to control in CC. In SMD a*β*42 (*p* < 0.0001), APP (*p* < 0.002) compared to SMD control, and compared to CC control, in SMD control flies showed a significant increase in a*β*42 (*p* < 0.0001). a*β*42 object area (S.I. Figure 9E) increased in a*β*42 (*p* < 0.003), and APP (*p* < 0.0001) flies compared to CC control. In SMD, both a*β*42 and APP (*p* < 0.0001) showed a significant increase in a*β*42 object count compared to control.

### 3.2. Discussion

AD has been characterized as a central nervous system disorder driven by amyloid-*β* plaques and hyperphosphorylated tau tangles.^53^ ^,^ ^54^ However, accumulating evidence indicates that AD is influenced by systemic processes, including peripheral immune responses, metabolic regulation, and gut-brain communication.^55‐57^ Our study provides experimental evidence to support this concept by demonstrating that depletion of the gut microbiome in a humanized *Drosophila* model of AD is associated with exacerbation of biochemical, molecular, cellular, and behavioral features of disease pathology. Comparisons between AD flies under CC and SMD conditions suggest that the gut microbiome may modify AD-associated phenotypes. Our study found an altered microbiome population in the gut of AD transgenic flies. *Drosophila* gut-abundant commensal microbiome *Lactobacillus* and *Acetobacter* ^43^ showed differences among the AD transgenic flies. These observations are consistent with human and murine studies reporting that AD-associated dysbiosis varies with disease stage, host genetics, and metabolic state. For example, alterations in *Lactobacillus, Bifidobacterium*, and pro-inflammatory taxa such as *Escherichia/Shigella* have been linked to increased peripheral inflammation and cognitive decline.^58^ ^,^ ^59^ Since the *Drosophila* AD model recapitulates microbial shifts (Figure 1, S.I. Figure 1) like previous studies,^58^ ^,^ ^59^ the similarity between these microbial alterations and those reported in mammalian studies supports the use of *Drosophila* as a genetically tractable model for investigating gut-brain communication during AD pathology.

One of the fundamental observations of our study was the upregulation of proinflammatory cytokines and their downstream JAK/STAT signaling genes when the microbiome quantity diminished in SMD flies (Figure 3; S.I. Figure 3). In AD flies, we observed upregulation of the *Upd-Dome-Hop-Stat92e* axis, which is analogous to the mammalian IL−6/JAK/STAT signaling, implicated in AD-associated neuroinflammation, microglial activation^60^ ^,^ ^61^ and synaptic dysfunction in AD.^61^ To further examine whether enhanced glial-specific inflammatory signaling was associated with AD pathology, we analyzed protein expression changes in glial-UPD3 overexpression flies (S.I. Figure 8). We found increased levels of STAT5A (STAT92E) along with Tau, pTau, a*β*42, and APP proteins (SI. Figure 8). This finding aligns with our qPCR results, further highlighting the role of inflammatory signaling in AD pathology. Our findings support the potential association between microbiome depletion and activation of inflammatory signaling; however, further studies are required to establish the underlying mechanistic relationship and intermediates involved. Studies in mammalian models showed that microbial metabolites, specifically short-chain fatty acids (SCFAs) such as acetate and butyrate, suppress inflammatory responses driven by NF-kB and STAT signaling.^60^ ^,^ ^62^ Although our study did not measure SCFAs, previous mammalian studies suggest that microbiome depletion may reduce SCFA availability.^63^ A lack of microbially derived metabolites further exacerbates the inflammatory responses by reactive gliosis and reduces the clearance of protein aggregates,^60^ ^,^ ^64^ which further accelerates neuronal cell death. Our data is consistent with the upregulation of apoptotic mediators such as *Hid* and *Reaper* under SMD conditions in both head and body regions (Figure 3; S.I. Figure 3), supporting the idea that upregulation of inflammation directly impacts cell death pathways.^65^ ^,^ ^66^ These findings suggest that microbiomes contribute to maintaining an intricate balance between immune homeostasis, neuroinflammation, and apoptotic signaling, whereas their depletion may worsen AD-associated pathology.

Growing evidence supports a connection between AD and metabolic dysfunction, with strong association with disturbances in energy homeostasis, lipid dysregulation, mitochondrial dysfunction and protein quality control pathways.^67-69^ In AD, a consistent pathological signature includes increased *p*-AMPK, accumulation of p62 (Ref(2)P), and elevated levels of ubiquitinated proteins, reflecting impaired proteostasis. AMPK activation indicates chronic neuronal energy stress and promotes autophagy initiation,^48^ while Ref(2)p/p62, an adapter that binds ubiquitinated cargo, is normally degraded during autophagy and therefore accumulates when autophagic flux is defective.^70^ The concurrent increase in ubiquitinated protein aggregates and neurofibrillary tangles further suggests a possible failure in protein clearance systems.^71^ Our study further extends this view by showing increased lipid droplet accumulation and Ref(2)p/p62, reduced LC3 and LC3-II/I ratio following gut microbiome depletion in AD transgenic flies (Figures 2 and 6, S.I. Figure 7). Increased lipid droplets in the head and brain regions further support the possibly altered lipid homeostasis,^72^ ^,^ ^73^ and role for the gut microbiome.^74^ ^,^ ^75^ It also correlates with results observed in both murine models and human AD patients.^76^ Interestingly, SMD AD flies showed increased phosphorylation of AMPK, a master regulator that checks cellular energy balance.^48^ Collectively, the increase in *p*-AMPK, ubiquitinated proteins, Ref(2)p, and LC3-I with reduced LC3-II suggests a possible impairment in autophagic flux and defective protein degradation.^48^ ^,^ ^51^ ^,^ ^77^ ^,^ ^78^ Consistent with these findings, immunofluorescence analysis further demonstrated an increase in *β*-amyloid accumulation in SMD AD flies. Our findings support the notion that microbiome depletion may exacerbate AD-associated protein pathology through impaired protein clearance. Based on our study on SMD flies, we believe that one of the possible explanations is that microbiome depletion alters nutrient availability and microbial metabolite production,^46^ thereby promoting cellular energy stress and AMPK activation. Another possibility lies within the AMPK-dependent activation of the autophagy mechanism. AMPK regulates ULK1 under glucose starvation and amino acid starvation differently.^48^ Hence, we believe that energy stress conditions promote AMPK activation but fail to accomplish autophagy.^79^ Our findings further indicate the possible role of microbiome-derived metabolites involved in enhancing lysosomal and autophagy functions.^80-82^ Together, these findings indicate that autophagy is activated but does not efficiently degrade protein aggregates, leading to the accumulation of ubiquitinated proteins and contributing to neurodegeneration.^83^ ^,^ ^84^ Based on these insights, we believe that the gut microbiome may help to mitigate AD pathology by supporting both lipid and autophagic homeostasis. Depletion of the gut microbiome could suppress these protective inputs, leading to impaired proteostasis, neurofibrillary tangle formation, and amyloid accumulation.^85^

In our study, SMD AD flies exhibited significant sleep/circadian disruption compared to CC flies (Figure 4; S.I. Figure 4). Circadian disruption, altered eating behavior, and sleep disturbances are well-known clinical features observed in AD patients.^86^ ^,^ ^87^ Our data support an association between gut microbiome depletion and altered sleep/circadian behavior. Apart from that, the gut microbiome itself exhibits diurnal oscillations in microbial composition and metabolite production that could interact with the host’s internal circadian clock proteins.^88^ These observations further indicate the role of the gut microbiome in synchronizing the host circadian system. Previous studies report that microbial metabolites could act as zeitgebers, entraining peripheral clocks through AMPK, sirtuins, and nuclear hormone receptors.^89-92^ Loss of these cues may aggravate the circadian misalignment that was already established by tauopathy and amyloid pathology and aggravate metabolic and cognitive dysfunctions.^93^ Since AD pathology disrupts circadian rhythms, reshaping the gut microbiome,^94^ which in turn amplifies cognitive decline and circadian misalignment,^55^ overall, our findings support the concept of a bidirectional gut-brain axis.^55^ This vicious cycle may partly explain the progressive worsening of behavioral dysfunction observed in AD patients.

One of the major pathological associations of cognitive decline in the AD condition is synaptic loss.^95^ We have observed a significant reduction in synapsin in the SMD flies.^96^ Increased phalloidin staining revealed alterations in the actin cytoskeleton. F-actin is essential for maintaining synaptic structure and plasticity.^97^ These changes may further contribute to the cognitive impairment observed in SMD AD flies through olfactory preference and shock avoidance tests (Figure 5). These findings may reflect the loss of gut microbiome-derived metabolic and trophic support required to maintain synaptic integrity.^98^ SCFAs have been reported to promote synaptic protein expression and dendritic spine maturation.^99^ Dysbiosis could lead to decreased synaptic markers and even memory performance.^100^ Based on these reports, we believe that gut microbiomes may be involved in maintaining synaptic integrity and cognitive function. In addition, we have observed that all AD genotypes exhibited cognitive impairment during SMD conditions, and that microbiome depletion may influence memory impairments across different AD genotypes.

An important conceptual insight from this study is that microbiome depletion does not appear to act through a single pathway but rather influences interconnected immune, metabolic, and neuronal processes (Figure 7 G). Within this context, the gut microbiome may contribute to maintaining homeostasis across these systems. Its depletion is associated with a shift toward a more vulnerable state, characterized by enhanced inflammatory signaling, metabolic dysregulation, impaired proteostasis, and behavioral changes. These effects are unlikely to be independent and may instead interact with each other, potentially contributing to disease progression. This interpretation is broadly consistent with recent cross-species studies,^55^ ^,^ ^101^ which suggest that microbiome perturbations can modulate AD-related phenotypes through coordinated immune–metabolic interactions.

### 3.3. Limitations and future directions

Our study provides a comprehensive evaluation of microbiome depletion in glial-AD models. Some limitations remain to be addressed. Identification of specific microbial species and their metabolites involved in regulating glial function will provide a deep understanding of host-microbiome interactions and implications of neurodegeneration. Studying the inflammatory and proteostatic signaling mechanisms will provide a promising venue for mechanistic AD research progression. Microbiome reconstitution or targeted microbial intervention studies could provide more information regarding disease response to microbiome interventions. Extending these findings to higher organisms like animals and humans is essential to evaluate the therapeutic relevance of current findings. The present study establishes a strong experimental framework for future studies focused on understanding microbiome-dependent therapeutic strategies to treat neurodegenerative disorders.

### 3.4. Conclusion

The current manuscript discusses how microbiome depletion exacerbates glial-driven neuroinflammation, sleep/circadian disruption, and proteostasis imbalance in an AD model of *Drosophila*, which mainly highlights the gut microbiome as an important factor contributing to the neurodegenerative phenotype. Our study provides a mechanistic preclinical framework suggesting that metabolic homeostasis and glial resilience are highly influenced by microbial signals during AD progression. As our model system enables precise genetic manipulation of gut-brain interactions, future studies using mammalian systems will help to confirm the translational relevance of these pathways. We believe our study supports the concept that gut microbiome-glial interaction contributes to AD pathology and endorses a new direction for future studies in the context of neurodegenerative research.

## Supplementary Material

Supplemental Material Madamanchi_et_al_Supplementary_Material.docx

Source Data as Supplementary Material.xlsx Source Data as Supplementary Material.xlsx

## Data Availability

The authors confirm that data supporting the findings of the current study are provided in the manuscript, supplementary material, and source data file.

## ARRIVE checklist

All the guidelines have been followed, and specific information can be found in the attached arrive checklist form.
