# Supplementary material for: Gut microbiome depletion aggravates glial-mediated Alzheimer’s disease pathology through inflammatory, metabolic, and proteostatic dysregulation
Source: Gut Microbes. 2026 Nov 8;18(1):2753247. doi: 10.1080/19490976.2026.2753247 (PMC13757599; doi:10.1080/19490976.2026.2753247)
Supplement: Supplementary file 1 [file KGMI_A_2753247_SM9273.docx]

**File type: Supplementary information:**

**Gut Microbiome Depletion Aggravates Glial-Mediated Alzheimer’s Disease Pathology Through Inflammatory, Metabolic, and Proteostatic Dysregulation**

**Kishore Madamanchi^1^, Srinath Gurrala^1^, John Watson^1^, Girish Melkani^1*^**

**^1^University of Alabama at Birmingham.**

^2^UAB Nathan Shock Center, Birmingham, AL 35294

**Email:** [girishmelkani@uabmc.edu](mailto:girishmelkani@uabmc.edu)

Kishore Madamanchi

Orcid ID: 0000-0002-5968-5177

**Supplementary data:** A single file contains supplementary figures S1-S10 and figure legends. This supplementary section also contains raw data with uncut western blot images for the main text Figure. 6 and supplementary Figures 7 (from page 21 to 27).

**SI. Figure 1.** Represents the comparison of most abundant microbial population in fly gut between conventional control (CC) and axenic antibiotic (SMD represented with *) flies carrying AD transgenes expressed under *Glaz-Gal4* glial cell specific driver at 3 weeks age. (A). *Lactobacillus* genus SMD flies showed a significant reduction in Tau, pTau, a*β*42, APP (p<0.0001) and GFP, Tau*^R406W^* (p<0.01) compared to CC flies. (B). *L. brevis* showed significant decrease in SMD flies (p<0.0001) compared to CC flies in *Glaz*/+, Tau, pTau, a*β*42, and APP flies. (C). *L. plantarum* Significant reduction in GFP, Tau (p<0.001), Tau*^R406W^*, pTau, a*β*42, APP (p<0.0001) in SMD flies compared to CC. (D). *Acetobacter* genus in SMD flies showed a significant reduction in *Glaz*/+, GFP, Tau, pTau, a*β*42, APP (p<0.0001) and Tau*^R406W^* (p<0.01) compared to CC flies. (E). *A. pasteur* in *Glaz*/+, Tau, and APP flies in SMD showed significant decrease compared to CC flies. *A. promorum Glaz*/+, GFP (p<0.01) and Tau, Tau*^R406W^*, pTau, a*β*42, APP (p<0.0001) in SMD flies decreased significantly compared to CC flies. (G). *A. pasteurianus* showed significant reduction in GFP (p<0.01), Tau, Tau*^R406W^*, pTau, a*β*42, APP (p<0.0001) in SMD flies compared to CC flies. (H). 16 rRNA quantification showed significant reduction in GFP (p<0.01), Tau, pTau, a*β*42, APP (p<0.0001) in SMD flies compared to CC flies. (I). *L. plantarum*, not showed any significant increase in head region, but in the body GFP (p<0.04), Tau*^R406W^*(p<0.006) and Tau, pTau, a*β*42 and APP (p<0.0001) showed significant increase compared to control flies. Compared to the head in the body region Tau, Tau*^R406W^*, pTau, a*β*42 and APP (p<0.0001) showed a significant increase. (J). *L.brevis* did not show any significant increase in head region, but in the body Tau (P<0.02), pTau (p<0.004), and APP (p<0.0006) showed significant increase compared to head region. (K). *A. promorum,* not showed any significant increase in head region, but in the body Tau (p<0.0001) pTau (p<0.003), and a*β*42 (p<.0005) showed significant increase compared to control flies. Compared to the head in the body region Tau, pTau, a*β*42, APP (p<0.0001) and Tau*^R406W^* (p<0.003) showed a significant increase. (L). *A. pasteurianus* significantly increased in APP (p< 0.0001) compared to control in the head. In the body region Tau, pTau, a*β*42 (p<0.0001) and APP (p<0.0006) showed significant increase compared to control and compared to head Tau, pTau, a*β*42 (p<0.0001) and APP (p<0.02) showed significant increase compared to head region. (M). *Enterococcus* in the body Tau (p<0.0001) pTau (p<0.003), and a*β*42 (p<.0005) showed significant increase compared to control flies. Compared to the head in the body region Tau, pTau, a*β*42, APP (p<0.0001) and Tau*^R406W^* (p<0.003) showed a significant increase. . (N). *Leuconostoc*, in the body GFP (p<0.04), Tau*^R406W^*(p<0.006) and Tau, pTau, a*β*42 and APP (p<0.0001) showed significant increase compared to control flies. Compared to the head in the body region Tau, Tau*^R406W^*, pTau, a*β*42 and APP (p<0.0001) showed a significant increase. (O). *Gluconobacter* SMD flies showed a significant reduction in Tau, pTau, a*β*42, APP (p<0.0001) and GFP, Tau*^R406W^* (p<0.01) compared to CC flies. (B). *L. brevis* showed significant decrease in SMD flies (p<0.0001) compared to CC flies in *Glaz*/+, Tau, pTau, a*β*42, and APP flies. (P). *Providencia* Significant reduction in GFP, Tau (p<0.001), Tau*^R406W^*, pTau, a*β*42, APP (p<0.0001) in SMD flies compared to CC. (Q). *Enterobacter* in SMD flies showed a significant reduction in *Glaz*/+, GFP, Tau, pTau, a*β*42, APP (p<0.0001) and Tau*^R406W^* (p<0.01) compared to CC flies. (R). *Pseudomonas* in *Glaz*/+, Tau, and APP flies in SMD showed significant decrease compared to CC flies. *A. promorum Glaz*/+, GFP (p<0.01) and Tau, Tau*^R406W^*, pTau, a*β*42, APP (p<0.0001) S.I. Figure. 1 (A-H & M-R), One-way ANOVA was, with Šídák's multiple comparisons test as a post-hoc test was used to compare CC and SMD flies in the body region. S.I. Figure. 1 (I-L) Two-way ANOVA with Šídák's multiple comparisons test as a post-hoc test was used to compare head and body regions. Blue asterisks represent the comparison between Glaz/+ and AD genotypes in each condition and red asterisks represent the comparison between identical genotypes reared under CC and SMD conditions. n=3 biological replicates in every genotype and condition (each replicate consists of 8-10 flies), and p-values * <0.05, ** <0.01, *** < 0.001, **** <0.0001. Raw data and specific p values are provided in the source data file.

**S.I. Figure 2.** The immunofluorescence images were repressing the individual channels for DAPI (White), Lipid spot (Orange), synapsin (Magenta), and phalloidin (Green): Figure. 2 representative images were separated into individual channels and provided as S.I. Figure. 2, which contains head and abdomen regions of all representative images.

**SI. Figure 3**. Represents cytokine (*Upd*1, and 2), *Eiger* (*TNFα* homolog) its downstream target *Imd* and cell death marker *Reaper* expression in head and body regions. (A). *Upd*1 expression has no significant difference in CC flies, in SMD Tau*^R406W^*, and APP (p<0.0001) showed a significant difference compared to controls. Tau (p<0.01), Tau*^R406W^*, and APP (p<0.0001) showed significant increase compared to CC flies. (B). *Upd*2 expressions found to be increased only in Tau*^R406W^* (p<0.0001) compared to control and CC counterpart in SMD flies. (C). *Eiger,* showed increased expression in Tau*^R406W^* (p<0.0001), and APP (p<0.1) compared to control in SMD flies and Tau*^R406W^* (p<0.0001) compared to CC flies. (D). *Imd* increase in GFP (p<0.01), Tau*^R406W^* (p<0.0001), GFP (p<0.001), Tau*^R406W^* (p<0.0001), and APP (p<0.01) compared CC flies. (E). *Reaper* gene expression increased in SMD Tau*^R406W^*, APP (p<0.0001), and pTau (p<0.05) compared controls and Tau*^R406W^* (p<0.0001), APP (p<0.01) compared to CC flies in head region. (F). Schematic representation of inflammatory signaling through *Upd 3-Stat92e* pathway. (G). *Upd* 1 expression decreased in Tau, pTau and APP (p<0.01) in CC flies, and Tau (p<0.05), pTau, APP (p<0.0001) and a*β*42 (p<0.001) in SMD flies compared to controls. In SMD flies Tau, pTau, a*β*42 and APP (p<0.0001) showed significant increase compared to CC flies. (H). *Upd 2* showed significant decrease in Tau (p<0.01) in CC flies, a*β*42 and APP (p<0.0001) in SMD flies compared to controls. In SMD flies Tau, a*β*42 and APP (p<0.0001) showed a significant increase compared to CC flies. (I). *Eiger* expression increased in pTau (p<0.05), a*β*42 (p<0.001) and APP (p<0.0001) compared to control in SMD flies and Compared to CC flies GFP (p<0.01), Tau, pTau (p<0.001), a*β*42 and APP (p<0.0001) increased significantly in SMD flies. (J). Imd upregulated in SMD Tau (p<0.05) and APP (p<0.0001) compared to control, also Tau, APP (p<0.0001), Tau*^R406W^* (p<0.001), and pTau (p.0.05) showed significant increase in SMD flies compared to CC flies. (K). *Reaper* significantly upregulated in SMD, Tau, Tau*^R406W^*, pTau, APP (p<0.0001) and a*β*42 (p<0.01) compared to controls. Also, compared to CC flies Tau (p<0.001), Tau*^R406W^*, pTau, APP (p<0.0001) and a*β*42 (p<0.01) increased significantly in SMD flies. Blue asterisks represent the comparison between Glaz/+ and AD genotypes in each condition and red asterisks represent the comparison between identical genotypes reared under CC and SMD conditions. n=3 biological replicates in every genotype and condition (each replicate consists of 8-10 flies), and p-values * <0.05, ** <0.01, *** < 0.001, **** <0.0001. Raw data and specific p values are provided in the source data file.

**SI. Figure 4.** Represents the bout numbers, and bout length. in CC and SMD flies at 3 weeks age. (A). Day sleep bout numbers increased in APP flies compared to control in CC, and in SMD flies GFP, Tau (p<0.01) and a*β*42 (p<0.0001) compared to controls, (B). Night sleep bout numbers increased in pTau and APP (p<0.05) in CC flies, In SMD flies GFP (p<0.05), Tau*^R406W^* (p<0.01) compared to controls. In APP of SMD flies significant increase observed (p<0.0001) compared to CC flies. (C). Total sleep bout numbers increase in a*β*42, APP (p<0.0001) of CC flies, in SMD flies GFP, pTau (p<0.0001) and Tau (p<0.001) compared to controls. In APP of SMD flies significant increase observed (p<0.001) compared to CC flies. (D) Day sleep bout length decreased in GFP, Tau, and pTau (p<0.01), Tau*^R406W^* (p<0.001) and APP (p<0.0001) compared to controls. In SMD flies GFP, Tau*^R406W^* (p<0.0001), Tau, a*β*42 (p<0.01) decreased significantly compared to control. In SMD flies Tau*^R406W^* (p<0.0001) significantly decreased, and APP (p<0.0001) significantly increased compared to CC flies. (E). Night sleep bout length decreased in Tau, pTau (p<0.05), Tau*^R406W^* , a*β*42 (p<0.01) and APP (p<0.0001) in CC flies compared to control. In SMD flies GFP, Tau, Tau*^R406W^* (p<0.0001), and pTau (p<0.01) decreased compared to controls. In SMD GFP (p<0.01), decreased and a*β*42 (p<0.001), APP (p<0.0001) significantly increased compared to CC flies. (F). Total sleep bout length Decreased in Tau, APP (p<0.0001), Tau*^R406W^* (p<0.001), and pTau (p<0.01) compared to control I n CC flies. In SMD flies GFP, Tau*^R406W^* (p<0.0001), and Tau (p<0.01) decreased significantly compared to control in SMD flies. In Tau*^R406W^* (p<0.001) decreased significantly and in APP (p<0.0001) increased significantly compared to CC flies. Blue asterisks represent the comparison between Glaz/+ and AD genotypes in each condition and red asterisks represent the comparison between identical genotypes reared under CC and SMD conditions. n=3 biological replicates in every genotype and condition (each replicate consists of 8-10 flies), and p-values * <0.05, ** <0.01, *** < 0.001, **** <0.0001. Raw data and specific p values are provided in the source data file.

**SI. Figure 5.** Male fly geotaxis performance during CC and SMD condition from 0 to 9 seconds. Climbing distance showed no significant difference between SMD and CC control flies. Whereas in SMD flies GFP and Glaz/+ showed reduced (p<0.0001, p<0.001) compared CC GFP. Tau found no significant difference. Tau*^R406W^* in CC showed a significant increase (p<0.0001, p<0.0001) compared to Glaz/+ and SMD Tau*^R406W^*. pTau in CC showed increased compared to SMD and Glaz/+ (p<0.03, p<0.001). a*β*42 in SMD showed a significant increase (p<0.001, p<0.008) compared to Aβ42 and Glaz/+ in CC. We also seen significant difference between SMD Glaz/+ and SMD a*β*42 (p<0.04). In APP, SMD flies showed a significant increase (p<0.0001, p<0.0001) compared to SMD, Glaz/+ and CC APP, in addition significant difference observed between CC Glaz/+ and CC APP (p<0.007). One-way ANOVA followed by Sidak’s multiple comparisons test used for statistical analysis. Black asterisks represent the comparison between Glaz/+ and AD genotypes in each condition and red asterisks represent the comparison between identical genotypes reared under CC and SMD conditions. n=3 biological replicates in every genotype and condition (each replicate consists of 11 flies), and p-values * <0.05, ** <0.01, *** < 0.001, **** <0.0001. Raw data and specific p values are provided in the source data file.

**SI. Figure 6.** Female fly geotaxis performance during CC and SMD condition from 0 to 9 seconds. Climbing distance showed significant difference (p<0.02) between SMD and CC control (Glaz/+) flies. In CC condition Glaz/+ showed significant increase (p<0.04) compared to GFP. In Tau CC showed significant increase compared to SMD (p<0.0001). Tau*^R406W^* in CC showed a significant increase (p<0.001, p<0.0001) compared to Glaz/+ and SMD Tau*^R406W^*. pTau in SMD showed significant increase compared to SMD Glaz/+ (p<0.0001). a*β*42 in CC showed a significant reduction (p<0.0001, p<0.0001) compared to Glaz/+ and SMD a*β*42. APP in CC showed a significant reduction (p<0.0001, p<0.0001) compared to Glaz/+ and SMD APP flies. One-way ANOVA followed by Sidak’s multiple comparisons test used for statistical analysis. Black asterisks represent the comparison between Glaz/+ and AD genotypes in each condition and red asterisks represent the comparison between identical genotypes reared under CC and SMD conditions. n=3 biological replicates in every genotype and condition (each replicate consists of 11 flies), and p-values * <0.05, ** <0.01, *** < 0.001, **** <0.0001. Raw data and specific p values are provided in the source data file.

**S.I. Figure. 7. Representative image of amyloid-*β* aggregates observed in AD-dysbiosis flies.** CC represents conventional control flies; SMD represents axenic antibiotic flies. **S.I. Figure 7A**. Amyloid-β aggregates tested using (AB1510) antibody and LC3 staining using GABARAP antibody. **S.I. Figure. 7B** showed quantification of amyloid-*β* aggregates band intensity, increased in pTau (p<0.001) and APP (p<0.0001) in SMD flies compared to control and pTau (p<0.05), APP (p<0.0001) compared to CC flies. **S.I. Figure. 7C** representing LC3-II/I ratio, which is decreased (p< 0.0001) in Tau, Tau*^R406W^*, pTau, and a*β*42 in CC flies compared to control. In SMD flies Tau*^R406W^* showed a significant reduction (p<0.03) compared to control. Between CC and SMD flies we found a significant reduction in Glaz/+, APP (p< 0.0008) and GFP (p<0.0001) compared to CC flies. All the representative blots (A) were shown along with internal control α-tubulin. Blue asterisks represent the comparison between Glaz/+ and AD genotypes in each condition and Red asterisks represent the comparison between identical genotypes reared under CC and SMD conditions. Statistical analysis performed with Two-way ANOVA to establish significance, followed by Sidak’s multiple comparisons test to assess the effects of genotype and microbiome condition. n=3, biological replicates in every genotype and condition (each replicate consists of 10-15 flies) and p-values * <0.05, ** <0.01, *** < 0.001, **** <0.0001. Raw data and specific p values are provided in the source data file.

**SI. Figure. 8. Represent the impact of over expression of inflammatory marker on AD pathology.** We have tested the inflammatory marker (UPD3 over expression fly line) to check whether UPD3 (cytokine IL6) alone can modulate AD associated proteins in the fly. In our study we have observed several AD and inflammatory markers. (A). represents western blot images. (B). presence of Amyloid beta UPD3 over expression (OE) line compared to control (p<0.02). (C). APP is also found to be increased in OE line (p<0.03), (D). Tau protein is significantly increased (p<0.004), (E). pTau also increased (p<0.005) in UPD3 OE model, further STAT5A (Stat92e), a downstream target was also studied, which showed significantly high (p<0.002) in the OE line compared to controls. All the representative blots (A) were shown along with internal control α-tubulin. One-way ANOVA followed by Dunnett's multiple comparisons test used for statistical analysis. Black asterisks represent the comparison between Glaz/+ and experimental groups. Statistical analysis performed with n=3, biological replicates in every genotype and condition (each replicate consists of 10-15 flies) and p-values * <0.05, ** <0.01, *** < 0.001, **** <0.0001. Raw data and specific p values are provided in the source data file.

**SI. Figure. 9. Depletion of microbiome modulating a*β*42** **and APP protein aggregation in abdomen region of CC and SMD flies.** Immunofluorescence analysis for CC, SMD flies carrying AD transgenes (pTau, a*β*42and APP) showed a significant variation. (B). APP (Green) objects count show no difference in CC flies but found a positive trend in the SMD flies compared to CC. (C). APP object area in the brain showed significant uptrend in the SMD flies and a*β*42 showed a significant increase (p<0.02) compared to CC and SMD control. (D). a*β*42 object count increased in a*β*42, APP (p<0.0001) compared to control in CC. In SMD a*β*42 (p<0.0001), APP (p<0.002) compared to SMD control and compared to CC control in SMD control flies showed significant increase in a*β*42 (p<0.0001). (E). a*β*42 object area increased in a*β*42 (p<0.003), and APP (p<0.0001) flies compared to CC control. In SMD both a*β*42 and APP (p<0.0001) showed a significant increase in a*β*42 object count compared to control. We also observed a significant increase in APP (p<0.04) in a*β*42 object count compared to CC flies. Two-way ANOVA to establish significance, followed by Sidak’s multiple comparisons test to assess the effects of genotype and microbiome condition. n=5 in every genotype and condition (3 tissue sections for fly), and p-values * <0.05, ** <0.01, *** < 0.001, **** <0.0001. Raw data and specific p values are provided in the source data file.

**S.I. Figure. 10: Summary Figure: Schematic representation of Work plan, and gut microbiome quantification using CC, SMD flies. Experimental procedures including real time PCR for inflammatory, cell death markers and microbiome quantification. immunofluorescence analysis for lipid, synapsin and phalloidin staining. Sleep activity analysis with *Drosophila* Activity monitoring System (DAMS). Memory/Olfaction assay with T-maze. Geotaxis analysis and western blot analysis. Figure represents axenic fly data, compared with control (blue arrow) and between groups (CC vs SMD, red arrow). Star represents significance. Up-arrow (Significant increase) Down-arrow (Significant decrease).**

**Raw Uncut Western Blot Images**

**Glaz driven expression aβ42 in the head in control and AD models under CC and SMD**

Glaz _AD_**SMD**_ Head

Glaz _AD_**CC**_ Head

Glaz/+

GFP

Tau

R406W

pTau

AΒ42

APP

Glaz/+

GFP

Tau

R406W

pTau

AΒ42

APP

**Glaz driven expression Tau in the head in control and AD models under CC and SMD**

Glaz _AD_**CC**_ Head

Glaz _AD_**SMD**_ Head

Glaz/+

GFP

Tau

R406W

pTau

AΒ42

APP

Glaz/+

GFP

Tau

R406W

pTau

AΒ42

APP

**Glaz driven expression P-Tau in the head in control and AD models under CC and SMD**

Glaz _AD_**CC**_ Head

Glaz _AD_**SMD**_ Head

Glaz/+

GFP

Tau

R406W

pTau

AΒ42

APP

Glaz/+

GFP

Tau

R406W

pTau

AΒ42

APP

**Glaz driven expression Synaptotagmin 1 in the head in control and AD models under CC and SMD**

Glaz _AD_**CC**_ Head

Glaz _AD_**SMD**_ Head

Glaz/+

GFP

Tau

R406W

pTau

AΒ42

APP

Glaz/+

GFP

Tau

R406W

pTau

AΒ42

APP

**Glaz driven expression AMPK in the head in control and AD models under CC and SMD**

Glaz _AD_**SMD**_ Head

Glaz _AD_**CC**_ Head

Glaz/+

GFP

Tau

R406W

pTau

AΒ42

APP

Glaz/+

GFP

Tau

R406W

pTau

AΒ42

APP

**Glaz driven expression p-AMPK in the head in control and AD models under CC and SMD**

Glaz _AD_**CC**_ Head

Glaz _AD_**SMD**_ Head

Glaz/+

GFP

Tau

R406W

pTau

AΒ42

APP

Glaz/+

GFP

Tau

R406W

pTau

AΒ42

APP

**Glaz driven expression Ubiquitin in the head in control and AD models under CC and SMD**

Glaz _AD_**SMD**_ Head

Glaz _AD_**CC**_ Head

Glaz/+

GFP

Tau

R406W

pTau

AΒ42

APP

Glaz/+

GFP

Tau

R406W

pTau

AΒ42

APP

**Glaz driven expression Ref(2)p (p62) in the head in control and AD models under CC and SMD**

Glaz/+

GFP

Tau

R406W

pTau

AΒ42

APP

Glaz/+

GFP

Tau

R406W

pTau

AΒ42

APP

Glaz _AD_**SMD**_ Head

Glaz _AD_**CC**_ Head

**Glaz driven expression LC3-I/II in the head in control and AD models under CC and SMD**

Glaz/+

GFP

HTau

R406W

pTau

AB42

APP

Glaz/+

GFP

HTau

R406W

pTau

AB42

APP

Glaz _AD_**CC**_ Head

LC3-I 16 kDa

LC3-II 14kDa

α-tubulin 51 kDa

Glaz/+

GFP

HTau

R406W

pTau

AB42

APP

Glaz/+

GFP

HTau

R406W

pTau

AB42

APP

Glaz _AD_**SMD**_ Head

LC3-I 16 kDa

LC3-II 14kDa

α-tubulin 51 kDa

**AD related proteins during UPD3 over expression in glial cells**

Tubulin

Glaz/+

GFP_OE

Upd3_OE

α-Tubulin

42kDa

Glaz/+

GFP_OE

Upd3_OE

AΒ42

6E10

4kDa

APP

Glaz/+

GFP_OE

Upd3_OE

APP

100kDa

pTau

Glaz/+

GFP_OE

Upd3_OE

pTau

52kDa

STAT5A

94kDa

Glaz/+

GFP_OE

Upd3_OE

Tau

Tau

52kDa

Glaz/+

GFP_OE

Upd3_OE

**Glaz driven expression *aβ*42 in the head in control and AD models under CC and SMD**

Glaz _AD_**CC**_ Head

Glaz _AD_**SMD**_ Head
